# Semi-Supervised Multi-View SVDD via Manifold-Regularized Dictionary Learning for Anomaly Detection

**DOI:** 10.3390/s26196107

**Published:** 2026-09-26

**Authors:** Yong Tang, Bo Liu, Yanshan Xiao

**Affiliations:** 1 School of Automation, Guangdong University of Technology, Guangzhou 510006, China; 2 School of Computers, Guangdong University of Technology, Guangzhou 510006, China

**Keywords:** dictionary learning, semi-supervised anomaly detection, multi-view learning, manifold regularisation, support vector data description

## Abstract

Anomaly detection becomes considerably harder when labels are scarce and the data are described by several heterogeneous views. A handful of labelled samples rarely delineates the normal region well, and the unlabelled pool is itself often contaminated by anomalies, so treating unlabelled data as normal and feeding them into a one-class boundary constraint injects incorrect supervision. We address this with SMDL-SVDD, a semi-supervised multi-view support vector data description (SVDD) framework built on dictionary representation learning. The guiding idea is to let unlabelled samples act at the level of representation rather than the SVDD boundary, so no class assumptions are imposed on them. SMDL-SVDD jointly learns a synthesis and an analysis dictionary in each view to obtain sparse representations, and constrains the SVDD hypersphere using only the small sets of labelled normal and labelled anomalous samples. A graph Laplacian regulariser over all training samples preserves the local manifold structure, while a cross-view consistency term on the unlabelled samples exploits their geometric distribution and shares information across views. The view-specific decision functions are then fused into a single anomaly score. We solve the joint problem by alternating convex search and analyse its convergence. Across 23 anomaly detection tasks derived from six public datasets, SMDL-SVDD gives the highest area under the receiver operating characteristic curve (AUC) on 21, improving the mean AUC by 8.87–10.62 percentage points over single-view one-class methods, by 5.10–7.64 points over representative semi-supervised detectors and by 4.11–5.24 points over multi-view one-class methods. Significance, noise, label-ratio, parameter, convergence, ablation and runtime studies confirm that the gains are stable; the ablation shows that manifold regularisation contributes substantially to the improvement and acts complementarily to the cross-view consistency constraint.

## 1. Introduction

Anomaly detection seeks out the patterns or behaviours in a dataset that deviate markedly from the majority of normal samples, and it remains one of the central problems in data mining and machine learning [1]. Since sensing, monitoring and information systems were deployed at scale, the task has found application in medical diagnosis [2], financial fraud identification [3], network intrusion detection [4] and industrial fault diagnosis, among others. Anomalies, however, occur rarely, are expensive to collect and annotate, and follow a distribution that is unknown a priori. Building a detector that is both discriminative and robust when anomalous samples are scarce is therefore still an open problem.

Existing detectors are commonly grouped into clustering-based, density-based, distribution-based and model-based families [1]. Among these, model-based one-class methods have become the dominant paradigm, since they model only the target (normal) class and require no prior assumptions about the anomaly distribution. Support vector data description (SVDD) [5] and one-class support vector machine (OCSVM) [6] are the two canonical formulations: the former encloses the normal samples in a minimum-volume hypersphere in feature space and flags whatever falls outside it, while the latter separates the target data from the origin with a maximum-margin hyperplane. Under suitable conditions, the two are equivalent. A number of refinements have followed, such as DW-SVDD [7], which introduces *k*-nearest-neighbour density weights so that the boundary is pulled towards high-density regions, and GL-SVDD [8], which combines global and local regularisation. Applied to realistic, complex data, though, these methods still run into three difficulties.

First, classical SVDD and most of its variants assume that the training set consists purely of normal samples and thus exploit supervision from a single class only [5,7]. Data collected in practice usually contain both a small number of labelled anomalies and a large volume of unlabelled samples. The former cannot support classifier training on their own, yet they carry precisely the discriminative information that delineates the anomaly boundary, and discarding them wastes an available supervisory signal. The latter, because annotation is costly, typically dominate the training set, and a purely one-class formulation has no mechanism for mining their distributional information. How to exploit all three kinds of samples jointly under label scarcity is therefore a key question.

Second, real data are often described by multiple views or heterogeneous feature sets that are at once consistent and complementary [9]. A single-view SVDD cannot draw on complementary cross-view information, whereas naively concatenating the views ignores structural differences between them and introduces redundancy and noise. Several multi-view one-class methods have been proposed in response. MS-SVDD [10] learns a projection matrix per view, maps all views into a shared low-dimensional subspace, and builds an SVDD there; mSVDD [11] uses a deep network to learn hyperspheres in several semantic subspaces. Most of these methods, however, are formulated for training sets containing normal samples only, and none of them brings the discriminative sparse representations of dictionary learning, a small set of labelled anomalies, a large unlabelled pool, and SVDD boundary modelling together in one framework.

Third, SVDD is sensitive to the quality of the input features: noisy and redundant dimensions distort the learned hypersphere and drive up both false alarms and misses [7,8]. Dictionary learning is an effective representation technique in this respect. Through a synthesis and an analysis dictionary, it converts samples into more discriminative sparse representations, suppressing noise and reducing dimensionality while retaining the structure that matters for discrimination [12,13]. This offers a route to better feature quality for multi-view anomaly detection, but its combination with multi-view, semi-supervised SVDD detection has received little attention.

Motivated by these observations, we propose a semi-supervised multi-view SVDD anomaly detection method based on dictionary learning (SMDL-SVDD). It rests on three ingredients: cooperative multi-view feature extraction, efficient use of semi-supervised data as a constraint, and the SVDD modelling of the normal region as the core objective. Dictionary representation learning, multi-view information fusion, manifold-regularised semi-supervised representation learning and SVDD boundary modelling are unified in a single optimisation problem. It should be stressed that the methodological character of this work is not a plain combination of the established techniques listed above but the separation of roles between boundary supervision from the labelled samples and representation-level exploitation of the unlabelled samples, so that unlabelled data that may contain latent anomalies can take part in representation learning without directly affecting the SVDD boundary constraint. Figure 1 gives an overview of the framework.

Our main contributions are as follows:

1. We propose a unified semi-supervised multi-view SVDD framework for anomaly detection. Synthesis- and analysis-dictionary learning, SVDD boundary modelling driven by a small number of labelled normal and anomalous samples, and multi-view information fusion are optimised jointly, so that representation learning and boundary learning reinforce one another rather than proceeding in isolation.
2. We design a representation-level mechanism through which unlabelled samples participate in learning. Rather than treating them as normal and subjecting them to the one-class boundary constraint, SMDL-SVDD assigns them no class label at all and lets them act through sparse coding, manifold regularisation and cross-view consistency. The manifold term exploits the local geometry of the training samples; the consistency term encourages the same unlabelled sample to be represented coherently across views. Together they use the distributional information in the unlabelled pool while keeping the risk of incorrect supervision low.
3. We derive an optimisation algorithm based on alternating convex search that updates the synthesis dictionary, the analysis dictionary, the sparse representations and the SVDD parameters in turn, and we analyse its convergence behaviour and time complexity.
4. We validate the method systematically on 23 anomaly detection tasks constructed from six public datasets. SMDL-SVDD achieves the highest AUC on 21 of them and improves consistently over both semi-supervised detectors and multi-view one-class methods. Significance testing, together with studies of noise sensitivity, label ratio, hyperparameters, convergence, ablation and training time, further characterises its effectiveness, robustness and stability.

The remainder of the paper is organised as follows: Section 2 reviews work on dictionary learning, SVDD and multi-view one-class classification. Section 3 presents SMDL-SVDD together with its optimisation algorithm, convergence analysis and complexity analysis. Section 4 reports the experimental setup and results. Section 5 concludes and outlines directions for future work.

## 2. Related Work

This paper studies anomaly detection under label scarcity and multi-view heterogeneity within the SVDD framework. We review three strands of related work: dictionary learning (Section 2.1), SVDD and its variants (Section 2.2), and multi-view one-class classification (Section 2.3).

### 2.1. Dictionary Learning

Dictionary learning [12,13] represents a sample as a sparse linear combination of atoms drawn from an overcomplete dictionary, which reduces dimensionality and suppresses redundancy and noise while preserving discriminative structure. Since K-SVD [14] and online dictionary learning [15], the idea has been applied widely in image classification, feature selection and pattern recognition. Depending on whether class labels are used during training, the methods split into supervised and unsupervised branches.

Supervised dictionary learning [13,16,17,18,19,20] incorporates class labels when learning the dictionary and the sparse codes in order to strengthen discriminability. Gangeh et al. [13] applied it to speech emotion recognition; Geng et al. [16] constructed a discriminative dictionary for SRAF insertion in lithographic layouts; Li et al. [17] added a representation constraint and a coding-correlation term to improve reconstruction; Wu et al. [18] proposed a dictionary learning-based crossmodal hashing scheme; Yankelevsky and Elad [19] combined graph regularisation to preserve local geometry; and Shrivastava et al. [20] developed multiple-kernel dictionary learning for weakly supervised classification. Such methods are strongly discriminative but depend on a comparatively large pool of annotated samples.

Unsupervised dictionary learning [21,22,23,24,25,26] dispenses with labels and carries lower computational and storage cost. Yan et al. [21] learned a shared dictionary through domain adaptation for unsupervised facial expression recognition; Yan et al. [22] introduced Laplacian regularisation to compensate for the missing labels; Xu and Wang [23] proposed a double-layer sparse representation; and Ding et al. [24], Fan et al. [25] and Parsa et al. [26] coupled dictionary learning with unsupervised feature selection under graph learning, joint learning and low-rank constraints respectively.

Beyond learning a synthesis dictionary alone, some studies additionally introduce an analysis dictionary [12]. The synthesis dictionary reconstructs a sample from its sparse code, whereas the analysis dictionary generates the sparse code directly from the sample, thereby avoiding the expensive per-sample sparse coding step. Pairing the two offers an efficient way to extract discriminative sparse features, yet its use in multi-view, semi-supervised SVDD anomaly detection remains limited. This gap is one of the starting points of the present work.

### 2.2. Support Vector Data Description

SVDD [5] is a kernel method widely used for one-class classification and anomaly detection. Given a training set $X=\{x_{1},x_{2},\ldots ,x_{l}\}$, SVDD maps the samples into a high-dimensional feature space through ϕ(·) and constructs a hypersphere of minimum volume that encloses the normal samples as tightly as possible. The primal problem reads

(1) $$\begin{array}{cc} \min_{R,o,\xi }\; & R^{2}+C\sum_{i=1}^{l}\xi_{i}\\ s.t.\; & {∥\phi (x_{i})-o∥}^{2}\leq R^{2}+\xi_{i},\;i=1,2,\ldots ,l\\ \; & \xi_{i}\geq 0,\;i=1,2,\ldots ,l \end{array}$$

where *R* and o are the radius and centre of the hypersphere, C>0 trades volume off against training error, and $\xi_{i}$ are slack variables. Inner products in the feature space are evaluated through a kernel function,

(2) $$K\left(x,x_{i}\right)=\phi \left(x\right)\cdot \phi \left(x_{i}\right).$$

Introducing Lagrange multipliers gives the dual of (1),

(3) $$\begin{array}{cc} \max\; & \sum_{i=1}^{l}\alpha_{i}K\left(x_{i},x_{i}\right)-\sum_{i=1}^{l}\sum_{j=1}^{l}\alpha_{i}\alpha_{j}K\left(x_{i},x_{j}\right)\\ s.t.\; & 0\leq \alpha_{i}\leq C,\;i=1,2,\ldots ,l\\ \; & \sum_{i=1}^{l}\alpha_{i}=1 \end{array}$$

in which $\alpha_{i}$ are the Lagrange multipliers and the samples with $\alpha_{i}>0$ are the support vectors. The KKT conditions express the centre as a linear combination of the support vectors,

(4) $$o=\sum_{i=1}^{l}\alpha_{i}\phi \left(x_{i}\right).$$

For a test sample y, the decision function is

(5) $$f\left(y\right)={∥\phi (y)-o∥}^{2}-R^{2},$$

and y is declared normal when f(y)≤0 and anomalous otherwise. Under suitable conditions, SVDD and OCSVM [6] yield equivalent decision boundaries [5]; OCSVM separates the normal samples from the origin with a maximum-margin hyperplane and controls the anomaly fraction through a parameter ν, and both need only normal samples to perform detection.

Several refinements aim at sharper discrimination. Lee et al. [27] proposed density-induced SVDD (DI-SVDD), which introduces a relative density measure to reflect the sample distribution; Cha et al. [7] assigned *k*-nearest-neighbour density weights so that the boundary shifts towards dense regions; Hu et al. [8] combined global and local regularisation (GL-SVDD) to characterise anomalies; and Azami et al. [28] converted SVDD outputs into probability estimates for outlier detection. Taken together, this line of work still targets single-view data and makes little use of the abundant unlabelled samples present in the training set, which motivates our unification of dictionary learning, semi-supervised sample use and multi-view fusion inside the SVDD framework.

### 2.3. Multi-View One-Class Classification

Real data are frequently described by several views that satisfy a consensus principle and a complementarity principle [9]: the former requires different views to produce decisions that agree as far as possible, the latter stresses that views supplement one another. Most multi-view learning addresses binary classification, and comparatively few methods target one-class settings. Those that do fall broadly into subspace-based and network-based groups.

Subspace-based methods map every view into a shared low-dimensional subspace and construct a one-class classifier there. Sohrab et al. [10] proposed multimodal subspace SVDD (MS-SVDD), learning a projection matrix per view; Gôlo et al. [29] embedded views into a shared subspace with a multimodal autoencoder; and Degerli et al. [30] applied the idea to early myocardial infarction detection. These methods mainly express consensus, and a single shared subspace with a single classifier struggles to capture what distinguishes one view from another.

Network-based methods learn one-class classifiers in several subspaces using deep models. Hu et al. [11] proposed multimodal deep SVDD (mSVDD), which maps text into multiple subspaces, learns a hypersphere in each and ties the views together with a similarity regulariser; Wang et al. [31] explored multi-view deep one-class classification systematically; and Lei et al. [32] proposed deep one-class crop classification (DOCC) for multimodal satellite imagery under a positive–unlabelled framework. Consensus again receives most of the attention, and complementarity is modelled only weakly.

To handle consensus and complementarity together, some authors have turned to learning using privileged information [33], that is, additional information available at training time but not at test time. Tang et al. [34] used it for multi-view binary SVMs, and Burnaev and Smolyakov [35] proposed OCSVM+ with privileged information for malware detection.

In summary, existing multi-view one-class methods have made clear progress in modelling consensus and complementarity, but two gaps remain. Most are built on the assumption that the training set contains normal samples only, so the discriminative and distributional information carried by a few labelled anomalies and many unlabelled samples goes unused; and few works bring the discriminative sparse representations of dictionary learning into multi-view one-class classification to strengthen feature expression and suppress noise and redundancy. SMDL-SVDD is designed to close both gaps by unifying dictionary representation learning, multi-view fusion, manifold-regularised semi-supervised representation learning and SVDD boundary modelling in one framework.

## 3. Semi-Supervised Multi-View SVDD Based on Dictionary Learning

Section 3.1 and Section 3.2 set out the objective function and its solution; Section 3.3 states the resulting algorithm. Section 3.4 and Section 3.5 then analyse convergence and time complexity.

### 3.1. Construction of the Objective Function

The training data are split into three parts: labelled normal samples form the set $S_{N}$, labelled anomalous samples form $S_{M}$, and the remaining, much larger body of unlabelled samples forms $S_{U}$. Let $D^{v}=\left\{d_{1}^{v},d_{2}^{v},\ldots ,d_{k}^{v}\right\}\in R^{d_{v}\times k_{v}}$ denote the synthesis dictionary and $P^{v}=\left\{p_{1}^{v},p_{2}^{v},\ldots ,p_{k}^{v}\right\}\in R^{k_{v}\times d_{v}}$ the analysis dictionary of view *v*, with $k_{v}$ the dictionary size. The synthesis dictionary carries out feature extraction; adding the analysis dictionary simplifies the learning task considerably and lowers model complexity. For *V* views the objective is

(6) $$\begin{array}{cc} \min_{\begin{array}{c} D^{v},P^{v},A_{N}^{v},A_{M}^{v},A_{U}^{v}\\ R_{v},\xi_{N}^{v},\xi_{M}^{v} \end{array}}\;\sum_{v=1}^{V}[ & {∥X_{N}^{v}-D^{v}A_{N}^{v}∥}_{F}^{2}+{∥X_{M}^{v}-D^{v}A_{M}^{v}∥}_{F}^{2}+{∥X_{U}^{v}-D^{v}A_{U}^{v}∥}_{F}^{2}\\ & +\beta \left({∥A_{N}^{v}∥}_{2,1}+{∥A_{M}^{v}∥}_{2,1}+{∥A_{U}^{v}∥}_{2,1}\right)\\ & +\gamma ({∥P^{v}X_{N}^{v}-A_{N}^{v}∥}_{F}^{2}+{∥P^{v}X_{M}^{v}-A_{M}^{v}∥}_{F}^{2}\\ & \;\;\;+{∥P^{v}X_{U}^{v}-A_{U}^{v}∥}_{F}^{2})\\ & +R_{v}^{2}+C(\sum_{i=1}^{n_{N}}\xi_{N}^{v}\left(i\right)+\sum_{i=1}^{n_{M}}\xi_{M}^{v}\left(i\right))\\ & +\tau \mathrm{tr}\left(P^{v}X^{v}L^{v}{\left(X^{v}\right)}^{T}{\left(P^{v}\right)}^{T}\right)]+\delta \sum_{v_{1}\neq v_{2}}^{V}{∥A_{U}^{v_{1}}-A_{U}^{v_{2}}∥}_{F}^{2}\\ s.t.\;\; & {∥P^{v}x_{N}^{v}\left(i\right)-o^{v}∥}^{2}\leq R_{v}^{2}+\xi_{N}^{v}\left(i\right),\;x_{N}\in S_{N}\\ & {∥P^{v}x_{M}^{v}\left(i\right)-o^{v}∥}^{2}\geq R_{v}^{2}-\xi_{M}^{v}\left(i\right),\;x_{M}\in S_{M}\\ & \xi_{N}^{v}\left(i\right)\geq 0,\;\xi_{M}^{v}\left(i\right)\geq 0\\ & {∥d^{v}\left(k\right)∥}_{2}^{2}\leq 1 \end{array}$$

Four remarks explain the individual terms:

1. For the labelled normal samples, ${∥X_{N}^{v}-D^{v}A_{N}^{v}∥}_{F}^{2}$ reconstructs $X_{N}^{v}$ and therefore measures reconstruction error, while ${∥P^{v}X_{N}^{v}-A_{N}^{v}∥}_{F}^{2}$ measures sparse coding error, with $P^{v}X_{N}^{v}$ approximating the sparse matrix $A_{N}^{v}$. The labelled anomalous and unlabelled samples are treated in the same way.
2. ${∥A_{N}^{v}∥}_{2,1}$, ${∥A_{M}^{v}∥}_{2,1}$ and ${∥A_{U}^{v}∥}_{2,1}$ regularise the three sparse matrices through the $l_{2,1}$ norm, with β weighting the penalty.
3. The constraint ${∥P^{v}x_{N}^{v}\left(i\right)-o^{v}∥}^{2}\leq R_{v}^{2}+\xi_{N}^{v}\left(i\right)$ keeps the projection $P^{v}x_{N}^{v}\left(i\right)$ of a labelled normal sample within distance $R_{v}$ of the centre $o^{v}$, and ${∥P^{v}x_{M}^{v}\left(i\right)-o^{v}∥}^{2}\geq R_{v}^{2}-\xi_{M}^{v}\left(i\right)$ pushes the projection of a labelled anomaly beyond that radius. The slack variables $\xi_{N}^{v}$ and $\xi_{M}^{v}$ allow a controlled amount of violation and guard against overfitting. Unlabelled samples enter no hypersphere constraint at all; they participate through sparse coding, projection consistency and the cross-view consistency term, and their normal or anomalous status is decided by the final decision function once training has finished.
4. In ${∥d^{v}\left(k\right)∥}_{2}^{2}\leq 1$, $d^{v}\left(k\right)$ is the *k*-th atom (column) of the synthesis dictionary $D^{v}$. Bounding the atom norms prevents scale imbalance among atoms, stabilises the numerics, and restricts the parameter space in a way that helps generalisation.

The term $\mathrm{tr}(P^{v}X^{v}L^{v}{\left(X^{v}\right)}^{T}{\left(P^{v}\right)}^{T})$ is the manifold regulariser [36]. In every view we build a *k*-nearest-neighbour similarity (adjacency) matrix *W* over all training samples, unlabelled ones included, and derive the graph Laplacian matrix *L* from it. The term is equivalent to penalising, with similarity as the weight, the difference between the representations of neighbouring samples after projection by the analysis dictionary, so that the discriminative projection stays smooth along the data manifold. Through this mechanism the geometric distribution of the unlabelled samples propagates into the projection, which realises semi-supervised discrimination in the sense of the manifold (geometric smoothness) assumption. Both the similarity matrix *W* and the graph Laplacian matrix *L* are built on all $n=n_{N}+n_{M}+n_{U}$ training samples, that is, on the sum of the labelled normal, labelled anomalous and unlabelled counts. The adjacency matrix *W* and the graph Laplacian matrix *L* are defined as

(7) $$W_{ij}^{v}=\left\{\begin{array}{cc} \exp\left(-\frac{{∥x_{i}^{v}-x_{j}^{v}∥}_{2}^{2}}{2\sigma^{2}}\right),\; & x_{j}^{v}\in N_{k}\left(x_{i}^{v}\right)\\ 0, & \mathrm{otherwise} \end{array}\right.$$

(8) $$L^{v}=\Theta^{v}-W^{v},\;\Theta_{ii}^{v}=\sum_{j=1}^{n}W_{ij}^{v}.$$

The last term of (6) enforces cross-view consistency on the unlabelled samples. It penalises, in the Frobenius norm, the discrepancy between the analysis-dictionary sparse representations that the same unlabelled sample receives in different views. This drives the views towards a common discriminative representation, strengthens consensus, damps the noise and bias of any single view, and supplies a coherent basis for the multi-view decision fusion that follows. The parameter δ controls how strongly the constraint acts.

### 3.2. Optimisation of the Objective Function

To solve (6) we split the problem, holding the other views fixed while solving for view *v*. Within each view, the synthesis dictionary, the analysis dictionary, the sparse codes and the SVDD hypersphere are solved independently; the views are coupled only through the cross-view consistency constraint on the unlabelled samples. After training, the view-specific decision functions are fused into a single classifier. The individual updates are derived below.

**(1) Update $A_{N}^{v}$, $A_{M}^{v}$ and $A_{U}^{v}$ with $D^{v}$, $P^{v}$ and $R_{v}$ fixed**

Using the differentiable equivalent form of the $l_{2,1}$ norm [37], we write ${∥A_{N}^{v}∥}_{2,1}=2\mathrm{tr}\left({A_{N}^{v}}^{T}\Lambda_{N}A_{N}^{v}\right)$, where $\Lambda_{N}$ is diagonal with entries $\Lambda_{Nii}=1/\left(2{∥A_{N}^{vi}∥}_{2}\right)$ and $A_{N}^{vi}$ is the *i*-th row of $A_{N}^{v}$. Dropping the terms that do not involve $A_{N}^{v}$ gives

(9) $${A_{N}^{v}}^{*}=\arg\min_{A_{N}^{v}}L\left(A_{N}^{v}\right),$$

where

(10) $$\begin{array}{cc} L(A_{N}^{v}) & ={∥X_{N}^{v}-D^{v}A_{N}^{v}∥}_{F}^{2}+\gamma {∥P^{v}X_{N}^{v}-A_{N}^{v}∥}_{F}^{2}+\beta {∥A_{N}^{v}∥}_{2,1}\\ \; & ={∥X_{N}^{v}-D^{v}A_{N}^{v}∥}_{F}^{2}+\gamma {∥P^{v}X_{N}^{v}-A_{N}^{v}∥}_{F}^{2}+2\beta \mathrm{tr}\left({A_{N}^{v}}^{T}\Lambda_{N}A_{N}^{v}\right). \end{array}$$

Since $\Lambda_{N}^{i}\neq 0$, setting $\partial L\left(A_{N}^{v}\right)/\partial A_{N}^{v}=0$ yields

(11) $${A_{N}^{v}}^{*}={\left({D^{v}}^{T}D^{v}+\gamma I+2\beta \Lambda_{N}\right)}^{-1}\left({D^{v}}^{T}X_{N}^{v}+\gamma P^{v}X_{N}^{v}\right),$$

with I the identity matrix. The diagonal matrix $\Lambda_{N}$ is refreshed from the current estimate according to

(12) $$\Lambda_{Nii}=\frac{1}{2{∥A_{N}^{vi}∥}_{2}}.$$

The optimum for $A_{M}^{v}$ follows in the same way:

(13) $${A_{M}^{v}}^{*}={\left({D^{v}}^{T}D^{v}+\gamma I+2\beta \Lambda_{M}\right)}^{-1}\left({D^{v}}^{T}X_{M}^{v}+\gamma P^{v}X_{M}^{v}\right).$$

Writing $Q=\left({D^{v}}^{T}D^{v}+\gamma I+2\beta \Lambda_{U}\right)+2\delta \left(V-1\right)I$, we obtain

(14) $${A_{U}^{v}}^{*}=Q^{-1}[\left({D^{v}}^{T}X_{U}^{v}+\gamma P^{v}X_{U}^{v}\right)+2\delta \sum_{v^{\prime }\neq v}A_{U}^{v^{\prime }}].$$

**(2) Update $P^{v}$ with $D^{v}$, $A^{v}$ and $R_{v}$ fixed**

By removing the terms independent of $P^{v}$,

(15) $${P^{v}}^{*}=\arg\min_{P^{v}}L\left(P^{v}\right),$$

where

(16) $$\begin{array}{cc} L(P^{v})= & \gamma {∥P^{v}X_{N}^{v}-A_{N}^{v}∥}_{F}^{2}+\gamma {∥P^{v}X_{M}^{v}-A_{M}^{v}∥}_{F}^{2}+\gamma {∥P^{v}X_{U}^{v}-A_{U}^{v}∥}_{F}^{2}\\ & +\tau \mathrm{tr}\left(P^{v}X^{v}L^{v}{\left(X^{v}\right)}^{T}{\left(P^{v}\right)}^{T}\right). \end{array}$$

Differentiating with respect to $P^{v}$ and setting $\partial L\left(P^{v}\right)/\partial P^{v}=0$ give the optimum

(17) $$\begin{array}{cc} {P^{v}}^{*}= & \gamma \left(A_{N}^{v}{X_{N}^{v}}^{T}+A_{M}^{v}{X_{M}^{v}}^{T}+A_{U}^{v}{X_{U}^{v}}^{T}\right)\\ & \times {\left(\gamma X_{N}^{v}{X_{N}^{v}}^{T}+\gamma X_{M}^{v}{X_{M}^{v}}^{T}+\gamma X_{U}^{v}{X_{U}^{v}}^{T}+\tau X^{v}L^{v}{\left(X^{v}\right)}^{T}\right)}^{-1}. \end{array}$$

**(3) Update $D^{v}$ with $P^{v}$, $A^{v}$ and $R_{v}$ fixed**

By removing the terms independent of $D^{v}$,

(18) $$\begin{array}{cc} \min_{D^{v}} & {∥X_{N}^{v}-D^{v}A_{N}^{v}∥}_{F}^{2}+{∥X_{M}^{v}-D^{v}A_{M}^{v}∥}_{F}^{2}+{∥X_{U}^{v}-D^{v}A_{U}^{v}∥}_{F}^{2}\\ s.t. & {∥d^{v}\left(k\right)∥}_{2}^{2}\leq 1. \end{array}$$

To handle the norm inequality on each atom, we attach a non-negative Lagrange multiplier $\lambda_{k}\geq 0$$\left(k=1,\cdots ,k_{v}\right)$ to it, which gives the Lagrangian function

(19) $$\begin{array}{cc} L(D^{v})= & {∥X_{N}^{v}-D^{v}A_{N}^{v}∥}_{F}^{2}+{∥X_{M}^{v}-D^{v}A_{M}^{v}∥}_{F}^{2}+{∥X_{U}^{v}-D^{v}A_{U}^{v}∥}_{F}^{2}\\ & +\sum_{k=1}^{k_{v}}\lambda_{k}\left({∥d^{v}\left(k\right)∥}_{2}^{2}-1\right). \end{array}$$

By setting $\Lambda =\mathrm{diag}(\lambda_{1},\lambda_{2},\cdots ,\lambda_{k})$,

(20) $$\sum_{k=1}^{k_{v}}\lambda_{k}{∥d^{v}\left(k\right)∥}_{2}^{2}=\mathrm{tr}\left({D^{v}}^{T}D^{v}\Lambda \right),$$

and substituting (20) into (19),

(21) $$\begin{array}{cc} L(D^{v})= & {∥X_{N}^{v}-D^{v}A_{N}^{v}∥}_{F}^{2}+{∥X_{M}^{v}-D^{v}A_{M}^{v}∥}_{F}^{2}\\ & +{∥X_{U}^{v}-D^{v}A_{U}^{v}∥}_{F}^{2}+\mathrm{tr}\left({D^{v}}^{T}D^{v}\Lambda \right)-\mathrm{tr}\left(\Lambda \right). \end{array}$$

Differentiating with respect to $D^{v}$, setting $\partial L\left(D^{v}\right)/\partial D^{v}=0$ and rearranging give the closed-form optimum

(22) $${D^{v}}^{*}=\left(X_{N}^{v}{A_{N}^{v}}^{T}+X_{M}^{v}{A_{M}^{v}}^{T}+X_{U}^{v}{A_{U}^{v}}^{T}\right){\left(A_{N}^{v}{A_{N}^{v}}^{T}+A_{M}^{v}{A_{M}^{v}}^{T}+A_{U}^{v}{A_{U}^{v}}^{T}+\Lambda \right)}^{-1}.$$

Following Li et al. [12], we replace Λ with a global regularisation term εG, which removes the need to solve for Λ at every iteration and reduces the inversion to a single closed-form computation, lowering complexity and improving numerical stability at the same time. G is normally taken to be the identity matrix I, and ε≥0 is the global regularisation coefficient. Equation (22) then becomes

(23) $${D^{v}}^{*}=\left(X_{N}^{v}{A_{N}^{v}}^{T}+X_{M}^{v}{A_{M}^{v}}^{T}+X_{U}^{v}{A_{U}^{v}}^{T}\right){\left(A_{N}^{v}{A_{N}^{v}}^{T}+A_{M}^{v}{A_{M}^{v}}^{T}+A_{U}^{v}{A_{U}^{v}}^{T}+\varepsilon G\right)}^{-1}.$$

**(4) Update $R_{v}$ and $o^{v}$ with $P^{v}$, $A^{v}$ and $D^{v}$ fixed**

By removing the terms independent of $R_{v}$,

(24) $$\begin{array}{cc} \min_{R_{v},o^{v},\xi } & \sum_{v=1}^{V}\left[R_{v}^{2}+C\left(\sum_{i=1}^{n_{N}}\xi_{N}^{v}\left(i\right)+\sum_{i=1}^{n_{M}}\xi_{M}^{v}\left(i\right)\right)\right]\\ s.t. & {∥P^{v}x_{N}^{v}\left(i\right)-o^{v}∥}^{2}\leq R_{v}^{2}+\xi_{N}^{v}\left(i\right),\;x_{N}\in S_{N}\\ & {∥P^{v}x_{M}^{v}\left(i\right)-o^{v}∥}^{2}\geq R_{v}^{2}-\xi_{M}^{v}\left(i\right),\;x_{M}\in S_{M}\\ & \xi_{N}^{v}\left(i\right)\geq 0,\;\xi_{M}^{v}\left(i\right)\geq 0. \end{array}$$

Problem (24) can be optimised through its Lagrangian dual.

**Theorem** **1.** *By introducing Lagrange multipliers, the dual of problem (24) is*

(25) $$\begin{array}{cc} \max & \sum_{i=1}^{m}\alpha_{i}^{v}K_{v}\left(i,i\right)-\sum_{i=1}^{m}\sum_{j=1}^{m}\alpha_{i}^{v}\alpha_{j}^{v}K_{v}\left(i,j\right)\\ s.t. & -C\leq \alpha_{i}^{v}\leq C,\;i=1,\cdots ,m,\;\sum_{i=1}^{m}\alpha_{i}^{v}=1 \end{array}$$

*where $m=n_{N}+n_{M}$ and $K_{v}\left(i,j\right)=\langle P^{v}x_{i}^{v},P^{v}x_{j}^{v}\rangle$ is an inner product. The multipliers satisfy $0\leq \alpha_{i}^{v}\leq C$ if $x_{i}\in S_{N}$ and $-C\leq \alpha_{i}^{v}\leq 0$ if $x_{i}\in S_{M}$. The centre $o^{v}$ and radius $R_{v}$ of the hypersphere are given by*

(26) $$o^{v}=\sum_{i=1}^{m}\alpha_{i}^{v}P^{v}x_{i}^{v}$$

(27) $$R_{v}=\sqrt{K_{v}\left(a,a\right)+\sum_{i,j}\alpha_{i}^{v}\alpha_{j}^{v}K_{v}\left(i,j\right)-2\sum_{i}\alpha_{i}^{v}K_{v}\left(i,a\right)}$$

*where $x_{a}^{v}$ is a support vector lying on the hypersphere.*

**Proof.**  Attach non-negative Lagrange multipliers $\lambda_{N}^{v}\left(i\right)$, $\lambda_{M}^{v}\left(i\right)$ to the non-negativity constraints on the slack variables and $\eta_{N}^{v}\left(i\right)$, $\eta_{M}^{v}\left(i\right)$ to the two groups of hypersphere constraints in (24) for normal and anomalous samples respectively. This gives the Lagrangian function

(28) $$\begin{array}{cc} L= & R_{v}^{2}+C\left(\sum_{i=1}^{n_{N}}\xi_{N}^{v}\left(i\right)+\sum_{i=1}^{n_{M}}\xi_{M}^{v}\left(i\right)\right)-\sum_{i=1}^{n_{N}}\lambda_{N}^{v}\left(i\right)\xi_{N}^{v}\left(i\right)-\sum_{i=1}^{n_{M}}\lambda_{M}^{v}\left(i\right)\xi_{M}^{v}\left(i\right)\\ & -\sum_{i=1}^{n_{N}}\eta_{N}^{v}\left(i\right)\left(R_{v}^{2}+\xi_{N}^{v}\left(i\right)-{∥P^{v}x_{N}^{v}\left(i\right)-o^{v}∥}^{2}\right)\\ & -\sum_{i=1}^{n_{M}}\eta_{M}^{v}\left(i\right)\left({∥P^{v}x_{M}^{v}\left(i\right)-o^{v}∥}^{2}-R_{v}^{2}+\xi_{M}^{v}\left(i\right)\right). \end{array}$$

At the optimum, the partial derivatives of *L* with respect to the primal variables $R_{v}$, $o^{v}$, $\xi_{N}^{v}\left(i\right)$ and $\xi_{M}^{v}\left(i\right)$ all vanish. These stationarity (KKT) conditions give

(29) $$\sum_{i=1}^{n_{N}}\eta_{N}^{v}\left(i\right)-\sum_{i=1}^{n_{M}}\eta_{M}^{v}\left(i\right)=1$$

(30) $$o^{v}=(\sum_{i=1}^{n_{N}}\eta_{N}^{v}\left(i\right)P^{v}x_{N}^{v}\left(i\right)-\sum_{i=1}^{n_{M}}\eta_{M}^{v}\left(i\right)P^{v}x_{M}^{v}\left(i\right))$$

(31) $$\frac{\partial L}{\partial \xi_{N}^{v}\left(i\right)}=C-\lambda_{N}^{v}\left(i\right)-\eta_{N}^{v}\left(i\right)=0\;\Rightarrow \;\eta_{N}^{v}\left(i\right)\leq C$$

(32) $$\frac{\partial L}{\partial \xi_{M}^{v}\left(i\right)}=C-\lambda_{M}^{v}\left(i\right)-\eta_{M}^{v}\left(i\right)=0\;\Rightarrow \;\eta_{M}^{v}\left(i\right)\leq C.$$

Define a new variable $\alpha_{i}^{v}$ by setting $\alpha_{i}^{v}=\eta_{N}^{v}\left(i\right)$ for the normal samples $x_{N}^{v}\left(i\right)$, $i=1,\cdots ,n_{N}$, and $\alpha_{n_{N}+i}^{v}=-\eta_{M}^{v}\left(i\right)$ for the anomalous samples $x_{M}^{v}\left(i\right)$, $i=1,\cdots ,n_{M}$:

(33) $$\alpha_{i}^{v}=\eta_{N}^{v}\left(i\right),\;\alpha_{n_{N}+i}^{v}=-\eta_{M}^{v}\left(i\right).$$

Equation (29) then becomes

(34) $$\sum_{i=1}^{m}\alpha_{i}^{v}=1,$$

Equation (30) becomes

(35) $$o^{v}=\sum_{i=1}^{m}\alpha_{i}^{v}P^{v}x_{i}^{v},$$

and Equations (31) and (32) become

(36) $$\left\{\begin{array}{cc} 0\leq \alpha_{i}^{v}\leq C, & x_{i}\in S_{N}\\ -C\leq \alpha_{i}^{v}\leq 0, & x_{i}\in S_{M}. \end{array}\right.$$

Substituting these stationarity conditions back into (28) and eliminating the primal variables reduce the objective to a quadratic form in α alone, which is the dual problem (25). $x_{a}^{v}$ satisfying $0<\left|\alpha_{i}^{v}\right|<C$,

(37) $$R_{v}^{2}={∥P^{v}x_{a}^{v}-o^{v}∥}^{2}=K_{v}\left(a,a\right)+\sum_{i,j}\alpha_{i}^{v}\alpha_{j}^{v}K_{v}\left(i,j\right)-2\sum_{i}\alpha_{i}^{v}K_{v}\left(i,a\right),$$

and taking the square root of (37) gives (27), which completes the proof.    □

### 3.3. Algorithm

Algorithm 1 summarises the procedure. Once the alternating updates have converged, the decision function of view *v* is

(38) $$f_{v}\left(x\right)={∥P^{v}x-o^{v}∥}^{2}-R_{v}^{2},$$

and the views are combined into a single classifier:

(39) $$F\left(x\right)=\mathrm{sign}\left(\sum_{v=1}^{V}f_{v}\left(x\right)\right).$$

A sample is declared normal when F(x)≤0 and anomalous when F(x)>0.
**Algorithm 1** Semi-supervised multi-view SVDD anomaly detection based on dictionary learning.**Require:** Labelled normal samples $x_{N}^{v}\in S_{N}$, labelled anomalous samples $x_{M}^{v}\in S_{M}$, unlabelled samples $x_{U}^{v}\in S_{U}$; dictionary size $k_{v}$; regularisation parameters β, γ, *C*, δ; manifold coefficient τ; number of neighbours *k*; number of views *V*.**Ensure:** Final classifier F(x).  1:Initialise $D^{v}$ and $P^{v}$ as random matrices of unit Frobenius norm; build the *k*-nearest-neighbour similarity graph of each view and compute its graph Laplacian matrix; set Λ=0 and t=0.  2:**while** not converged **do**  3:      t←t+1  4:      **for** v=1 **to** *V* **do**  5:            With $D^{v}$, $P^{v}$, $R_{v}$ fixed, update $A_{N}^{v}$, $A_{M}^{v}$, $A_{U}^{v}$ by (11), (13), (14)  6:            Update the diagonal matrix Λ by (12)  7:            With $D^{v}$, $A^{v}$, $R_{v}$ fixed, update $P^{v}$ by (17)  8:            With $P^{v}$, $A^{v}$, $R_{v}$ fixed, update $D^{v}$ by (23)  9:            With $P^{v}$, $A^{v}$, $D^{v}$ fixed, update $o^{v}$ and $R_{v}$ by (26) and (27)10:      **end for**11:**end while**12:Compute the decision function of each view by (38)13:Fuse the view-specific classifiers by (39)

### 3.4. Convergence Analysis

The algorithm optimises (6) within the alternating convex search (ACS) framework, and the analysis below follows [38]. Two theorems are used.

**Theorem** **2.** *If $B\subseteq R^{n\times m}$, f:B→R is bounded below, and the optimisation of each variable is solvable at every iteration, then the sequence ${\left\{f\left(z_{i}\right)\right\}}_{i\in t}$ generated by ACS converges monotonically.*

**Theorem** **3.** *Let $X\subseteq R^{n}$ and $Y\subseteq R^{m}$ be closed sets, and let f:X×Y→R be continuous. If the optimisation of each variable is solvable at every iteration, then the following apply:*

*1.*  *If the sequence ${\left\{z_{i}\right\}}_{i\in t}$ generated by ACS is contained in a compact set, it has at least one accumulation point.*
*2.*  For every accumulation point $z^{*}$ of ${\left\{z_{i}\right\}}_{i\in t}$, the following apply:
  *(a)*  *If the optimal solution for one variable is unique when the others are fixed, then all accumulation points are local optima with the same function value;*
  *(b)*  *If the optimal solution for one variable is unique when the others are fixed, then $\lim_{i\to \infty }∥z_{i+1}-z_{i}∥=0$, and the accumulation points form a compact continuum C.*

Theorem 2 establishes solvability and convergence, and Theorem 3 characterises the optima. We use both to analyse Algorithm 1. Let *f* denote the objective of (6). For steps 5 to 9, write $D^{v(t)}$, $P^{v(t)}$, $A^{v(t)}$, $o^{v(t)}$ and $R_{v}^{\left(t\right)}$ for the values of $D^{v}$, $P^{v}$, $A^{v}$, $o^{v}$ and $R_{v}$ after the update at iteration *t*, and set $Z^{\left(t\right)}=\left(D^{v(t)},P^{v(t)},A^{v(t)},o^{v(t)},R_{v}^{\left(t\right)}\right)$. Algorithm 1 then generates the sequence $\left\{Z^{\left(t\right)}\right\}$, and the convergence of SMDL-SVDD is characterised by the following lemmas.

**Lemma** **1.** *The sequence $\left\{f\left(Z^{\left(t\right)}\right)\right\}$ generated by Algorithm 1 converges monotonically.*

**Proof.**  Equations (11), (13) and (14) update $A_{N}^{v}$, $A_{M}^{v}$ and $A_{U}^{v}$, and hence $A^{v}$, with $D^{v}$, $P^{v}$ and $R_{v}$ fixed. Equation (17) updates $P^{v}$ with $D^{v}$, $A^{v}$ and $R_{v}$ fixed. Equation (23) updates $D^{v}$ with $P^{v}$, $A^{v}$ and $R_{v}$ fixed. Equations (26) and (27) update $o^{v}$ and $R_{v}$ with $P^{v}$, $A^{v}$ and $D^{v}$ fixed. Each of these update problems is a convex subproblem with a closed-form solution and is bounded below by zero. In particular, after the manifold regulariser is introduced, the subproblem in $P^{v}$ remains a convex quadratic in $P^{v}$, because the graph Laplacian matrix $L^{v}$ is positive semi-definite, and it retains a closed-form solution requiring a single inversion. Since the optimisation of each variable is solvable at every iteration, Theorem 2 implies that $\left\{f\left(Z^{\left(t\right)}\right)\right\}$ converges monotonically. □

**Lemma** **2.** *The sequence $\left\{Z^{\left(t\right)}\right\}$ generated by Algorithm 1 has at least one accumulation point, and all accumulation points are local optima of f with the same function value.*

**Proof.**  Suppose that ${∥A^{v}∥}_{F}\to \infty$ or ${∥P^{v}∥}_{F}\to \infty$; this would force $f(Z^{\left(t\right)})\to \infty$. Lemma 1 has already established that $f(Z^{\left(t\right)})$ converges monotonically, so $\left\{Z^{\left(t\right)}\right\}$ must be contained in a compact set and is bounded in the finite-dimensional space. Conclusion (1) of Theorem 3 then gives at least one accumulation point. The proof of Lemma 1 shows that every variable subproblem is convex with a closed-form solution, so the optimal solution for each variable is unique, and conclusion (2a) of Theorem 3 implies that all accumulation points are local optima with the same function value. □

**Lemma** **3.** *If every subproblem in Algorithm 1 has a unique optimal solution when the remaining variables are fixed, then the sequence $\left\{Z^{\left(t\right)}\right\}$ satisfies*

(40) $$\begin{array}{cc} \lim_{t\to \infty } & ∥D^{v(t+1)}-D^{v(t)}∥+∥P^{v(t+1)}-P^{v(t)}∥+∥A^{v(t+1)}-A^{v(t)}∥\\ \; & +∥o^{v(t+1)}-o^{v(t)}∥+∥R_{v}^{\left(t+1\right)}-R_{v}^{\left(t\right)}∥=0. \end{array}$$

### 3.5. Time Complexity Analysis

Because the model is identical across views, it suffices to analyse a single view. For view *v*, $D^{v}\in R^{d_{v}\times k_{v}}$ is the synthesis dictionary, $P^{v}\in R^{k_{v}\times d_{v}}$ the analysis dictionary and $X^{v}\in R^{d_{v}\times n}$ the multi-view data, where *v* is the number of views, *n* the number of samples, $d_{v}$ the feature dimension of view *v* and $k_{v}$ its dictionary size. In general $n>d_{v}>k_{v}>v$.

Algorithm 1 solves for each variable by alternating convex search. Steps 5 and 6 update the sparse coding matrices at a cost of $O(d_{v}k_{v}n)$. Steps 7, 8 and 9 update the analysis dictionary $P^{v}$, the synthesis dictionary $D^{v}$, and the centre $o^{v}$ and radius $R_{v}$ of the hypersphere at costs of $O(d_{v}^{2}n)$, $O(d_{v}k_{v}n)$ and $O(k_{v}n^{2})$ respectively. The overall time complexity is therefore $O\left(v(d_{v}^{2}n+k_{v}n^{2})\right)$.

## 4. Experiments

This section introduces the datasets, the baseline methods and the parameter settings, then compares the proposed method against the baselines and analyses the results. All experiments were run on a machine with a 2.6 GHz processor and 16 GB of memory.

### 4.1. Datasets and Partitioning

#### 4.1.1. Datasets

To assess the method under multi-view and label-scarce conditions, we experiment on six public datasets of two kinds: Handwritten, Caltech101-7 and OutdoorScene provide natural multiple views, whereas WDBC, Mushroom and Wine are single-view datasets from which views are constructed. The views of WDBC, Mushroom and Wine are obtained by grouping the original features in a structured way according to their measurement source or semantic attribute, and these three datasets therefore serve mainly as supplementary validation for the constructed multi-view setting. Table 1 summarises their statistics.

The datasets are described in turn below.

**Handwritten (Multiple Features)**. It includes handwritten digits from 0 to 9, as shown in Figure 2. It contains 2000 samples, 200 per class, described by six natural views: Fourier coefficients (FOU; 76 dimensions), profile correlations (FAC; 216), Karhunen–Love coefficients (KAR; 64), pixel averages (PIX; 240), Zernike moments (ZER; 47) and morphological features (MOR; 6). The dataset is a standard benchmark for multi-view learning, its views are strongly complementary, and it fits the setting of our method most closely. The ten classes are denoted Handwritten-0 to Handwritten-9.

**Caltech101-7**. This is the commonly used seven-class subset of Caltech101, with 1474 samples described by six natural views: Gabor (48 dimensions), wavelet moments (WM, 40), CENTRIST (254), HOG (1984), GIST (512) and LBP (928). As image multi-view data, it tests the method on high-dimensional heterogeneous visual features. Because the classes are severely imbalanced, only Motorbikes and Face, which have enough samples, serve as normal classes; the remaining classes enter the anomaly pool only. The Motorbikes class is denoted by Cal-1 and the Face class by Cal-2.

**OutdoorScene**. This is a dataset of real outdoor scene images, comprising 2688 images across eight natural scene categories, described by four heterogeneous natural visual views: GIST (512 dimensions), colour moments (CM, 432), HOG (256) and LBP (48). As real-scene image multi-view data, it likewise tests the method on high-dimensional heterogeneous visual features. Given the class imbalance, only the four categories with sufficient samples—open country, mountain, coast and tall building—are used as normal classes, while the remaining categories enter the anomaly pool for downsampling. The four are denoted by Scene-1, Scene-2, Scene-3 and Scene-4 respectively.

**WDBC (Wisconsin Diagnostic Breast Cancer)**. This is a breast cancer diagnostic data with 569 samples (357 benign and 212 malignant). The 30 features consist of the mean, standard error and worst value of ten base measurements, which divides naturally into three views of ten dimensions each, matching the original design. The benign class is denoted by WDBC-1 and the malignant class by WDBC-2.

**Mushroom**. It includes mushroom data with 8124 samples (4208 edible and 3916 poisonous). The 22 discrete attributes are one-hot encoded and then grouped by attribute semantics into five views (cap, gill, stalk, ring–spore and habitat), which tests the method on views constructed from discrete attributes. The class of edible mushrooms is denoted by Mushroom-1 and that of poisonous ones by Mushroom-2.

**Wine**. Wine data with 178 samples, three classes and 13 physicochemical features, grouped by feature semantics into three views (basic physicochemical, phenolic, colour and other). It serves as a small-scale supplementary validation. The three classes are denoted by Wine-1, Wine-2 and Wine-3.

#### 4.1.2. Partitioning

Since most of these datasets are multi-class classification data, they must first be recast as anomaly detection tasks and then partitioned in a semi-supervised manner. We use a three-level protocol: construction of the anomaly detection task, the training/test split, and the semi-supervised label split within the training set.

**Level 1: task construction**. For each dataset we follow [5] and build anomaly detection tasks by taking one class in turn as normal and the remaining classes as anomalous.

**Level 2: training/test split**. The constructed data are split in a ratio of 7:3 by stratified random sampling, 70% for training and 30% for testing. The test set contains both normal and anomalous samples and is used to compute the evaluation metric. Every task is repeated independently over 10 random splits, and we report the mean AUC ± standard deviation.

**Level 3: semi-supervised label split within the training set**. Inside the training set, the three-way sample setting of our method is applied, yielding the labelled normal set $S_{N}$, the labelled anomaly set $S_{M}$ and the unlabelled set $S_{U}$. Let ρ be the fraction of labelled normal samples (ρ=25%); we draw that fraction at random from the training normal samples and label them to form $S_{N}$, while the remaining normal samples have their labels hidden and join $S_{U}$. Labelled anomalies are drawn at random at a fraction $\rho_{a}$ of the training anomalies ($\rho_{a}=20\%$) and labelled to form $S_{M}$, with the rest joining $S_{U}$ with labels hidden. The unlabelled set $S_{U}$ therefore contains both leftover normal samples and anomalies whose labels have been withheld, which reproduces the realistic situation in which the unlabelled pool is contaminated by unlabelled anomalies. Throughout the paper this anomaly contamination of the unlabelled pool is kept terminologically distinct from the feature noise injected in Section 4.4.2, which perturbs the feature values themselves.

This protocol yields 23 learning tasks in total; Table 2 reports their statistics. Each row corresponds to one learning task in which the stated class is normal and the anomalous samples are obtained by randomly downsampling the remaining classes to *r* times the number of normal samples (r=10%; for Wine, whose sample size is very small, r=40%). The anomaly ratio is the number of anomalous samples divided by the total.

### 4.2. Baselines

We compare SMDL-SVDD with eight mainstream anomaly detection algorithms: the single-view one-class methods SVDD [5] and OCSVM [6], together with the SVDD variant DW-SVDD [7]; the multi-view one-class methods MS-SVDD [10] and mSVDD [11]; and the semi-supervised methods SSAD [39], DeepSAD [40] and AnoOnly [41].

1. **SVDD** is a kernel method for one-class classification and anomaly detection whose core idea is to build a minimum-volume hypersphere enclosing the target data in feature space, with slack variables improving robustness to anomalies present in the training set.
2. **OCSVM** maps the target data into a high-dimensional feature space through a kernel and separates it from the origin with a maximum-margin hyperplane, bounding the anomaly fraction with a parameter ν; novelty and anomaly detection are achieved from one-class samples alone.
3. **DW-SVDD** computes density weights for the target samples from their *k* nearest neighbours and introduces those weights into the SVDD objective, so that the description boundary shifts preferentially towards high-density regions. This addresses the neglect of the data density distribution in the original SVDD.
4. **SSAD** is the kernel SVDD-based semi-supervised anomaly detection method by Görnitz et al. [39], which uses labelled normal, labelled anomalous and unlabelled samples together within a kernel one-class framework.
5. **DeepSAD** is the deep semi-supervised anomaly detection method by Ruff et al. [40], which extends Deep SVDD to the semi-supervised setting and learns a compact representation of normality end to end from a few labelled anomalies and many unlabelled samples.
6. **AnoOnly** is the semi-supervised anomaly detection method by Zhou et al. [41]. It is implemented on top of DeepSAD as the base model: the explicit loss supervision on the normal side is weakened, while a batch normalisation layer is inserted before the network output layer so that the normal data take part in weakly supervised representation learning through the batch statistics.
7. **MS-SVDD** learns a dedicated projection matrix for each view, maps heterogeneous multi-view data into a single low-dimensional shared subspace, and trains an SVDD jointly in that subspace to obtain a compact hyperspherical boundary for multi-view one-class classification and anomaly detection.
8. **mSVDD** adopts multi-view hypersphere modelling: after extracting text features with a shared neural network, it learns several hyperspheres corresponding to different semantic subspaces in the same feature space and fuses them by attention, giving accurate one-class classification of heterogeneous text data.

### 4.3. Experimental Setup and Evaluation Metric

#### 4.3.1. Parameter Settings

SVDD, OCSVM, DW-SVDD, MS-SVDD, SSAD and our SMDL-SVDD all use the radial basis function (RBF) kernel,

(41) $$K\left(x_{i},x_{j}\right)=\exp\left(\frac{-{∥x_{i}-x_{j}∥}_{2}^{2}}{2\sigma^{2}}\right),$$

with σ selected from $\left[2^{-3},2^{3}\right]$. Because mSVDD, DeepSAD and AnoOnly learn non-linear feature representations through end-to-end network training, their networks already play the role of the RBF kernel, so no kernel function is introduced for them. The parameter *C* of SVDD, DW-SVDD and SMDL-SVDD is selected from $\left[2^{-3},2^{3}\right]$. For OCSVM, ν is selected from {0.1,0.2,…,0.9,1}. The parameter *K* of DW-SVDD is set from {1,3,5,7}. For MS-SVDD, η is selected from {0.1,0.2,…,0.5} and β from $\left[10^{-3},10^{3}\right]$. For mSVDD, λ is selected from $\left[10^{-3},10^{3}\right]$ and ν from {0.1,0.2,…,0.9,1}. For SSAD, $\eta_{u}$ and $\eta_{l}$ are selected from $\left[10^{-2},10^{2}\right]$, and for DeepSAD η is selected from the same range. AnoOnly is implemented with DeepSAD as its base model, so the base-model parameter η uses the same range $\left[10^{-2},10^{2}\right]$ as DeepSAD, and the loss weight $\lambda_{n}$ of the unlabelled samples is selected from $\left\{0,10^{-6},10^{-4},10^{-2}\right\}$. For SMDL-SVDD, β, γ, δ and τ are selected from $\left[10^{-3},10^{3}\right]$ and the number of neighbours *k* is fixed at 5. All parameters are tuned by five-fold cross-validation.

#### 4.3.2. Evaluation Metric

AUC is the metric normally used in anomaly detection. It quantifies the area under the receiver operating characteristic (ROC) curve, which characterises model performance by plotting the true-positive rate (TPR) against the false-positive rate (FPR). Here TPR=TP/(TP+FN), where TP is the number of instances that belong to the target class and are predicted as such and FN the number that belong to the target class but are predicted otherwise; FPR=FP/(FP+TN), where FP is the number of instances that do not belong to the target class but are predicted as target and TN the number correctly predicted as non-target. An AUC of 1/2 corresponds to random guessing, and values closer to 1 indicate better performance.

### 4.4. Experimental Results

#### 4.4.1. Performance Comparison

Table 3 reports the mean AUC and standard deviation of the nine methods across the 23 anomaly detection tasks, with the best result in each row in bold. SMDL-SVDD attains the highest AUC on 21 of the 23 tasks; it is not best only on Handwritten-0 and Scene-1. These two exceptions show that the proposed method does not hold a uniform advantage over every data distribution. Possible contributing factors include the complexity of the within-class distribution, the degree of consistency and complementarity between the different views, and differences in how well a given representation model fits the specific data distribution. The advantage of the proposed method is visible against each family of baselines.

Compared with the conventional single-view one-class methods SVDD, OCSVM and DW-SVDD, SMDL-SVDD raises the mean AUC by roughly 10.62, 10.22 and 8.87 percentage points respectively. On WDBC-1, for instance, the three baselines reach 0.7589, 0.7673 and 0.8092, while SMDL-SVDD reaches 0.9500; on Wine-3, SMDL-SVDD attains 0.9917, again clearly above all three. This indicates that with multi-view data, learning a boundary from a single representation cannot fully exploit the complementary information across views, whereas joint multi-view modelling yields a more effective description of the data.

Compared with the semi-supervised detectors SSAD, DeepSAD and AnoOnly, SMDL-SVDD raises the mean AUC by roughly 7.64, 5.64 and 5.10 percentage points. On Mushroom-1 and Mushroom-2, it attains 0.9869 and 0.9738, against 0.9011 and 0.9137 for SSAD, 0.9439 and 0.9672 for DeepSAD and 0.9319 and 0.9334 for AnoOnly, and comparable margins appear on several Handwritten tasks. Under identical label-scarce conditions, then, exploiting semi-supervised label information alone remains limited, and combining it with multi-view representation information improves detection further.

Compared with the multi-view one-class methods MS-SVDD and mSVDD, SMDL-SVDD raises the mean AUC by roughly 5.24 and 4.11 percentage points, and the advantage is particularly stable on Wine, Caltech101-7 and Handwritten. The AUC reaches 0.9786 on Wine-1 and 0.9917 on Wine-3, the two Caltech101-7 tasks reach 0.9924 and 0.9915, and among the ten Handwritten tasks, all but Handwritten-0 give the highest AUC. Since MS-SVDD and mSVDD already exploit multi-view information, this suggests that multi-view subspace or multi-hypersphere modelling by itself does not make full use of the data structure available under label scarcity and that the dictionary representation learning, manifold constraint on unlabelled samples and cross-view consistency modelling that we adopt complement it well.

Taken together, SMDL-SVDD gives the best detection result on the large majority of the tasks and shows a consistent advantage against all three families of baselines. We therefore attribute the improvement to three mechanisms acting together: multi-view discriminative dictionary learning helps to extract more discriminative sparse representations from the raw features of each view; manifold regularisation over the unlabelled samples exploits the latent geometry of the data; and the cross-view consistency constraint promotes information sharing between views. They jointly support the detection capability of the model under label scarcity and multi-view heterogeneity.

To assess whether the improvements of SMDL-SVDD over the baselines are statistically significant, we follow the recommendation of [42] and run a Wilcoxon signed-rank test between SMDL-SVDD and each baseline; the results are shown in Table 4. In the table, $R^{+}$ and $R^{-}$ are the rank sums for which SMDL-SVDD is better and worse than the compared method, and a difference is regarded as significant when the *p*-value falls below the confidence level of 0.05. For all eight baselines, $R^{+}$ is far larger than $R^{-}$, and every *p*-value lies well below 0.05, so the gains of SMDL-SVDD over the various baselines are statistically significant.

#### 4.4.2. Sensitivity to Noise in the Data

In practice, data acquisition is affected by sensor error, measurement bias, transmission interference and missing samples, so observed samples inevitably carry random noise. To examine the robustness of SMDL-SVDD under noisy conditions, we inject Gaussian noise into the training set, following [43,44]. Specifically, we compute the standard deviation $\sigma_{i}^{0}$ along the *i*-th dimension of the training data and draw the noise standard deviation for that dimension at random from $\left[0,2\times \sigma_{i}^{0}\right]$, so that noise strength varies across dimensions. The feature-noise injection ratio is set to 0%, 10%, 20%, 30% and 40%, and at each level we compare the mean AUC of SMDL-SVDD against the baselines. We show six tasks chosen from datasets of different type and scale—Handwritten-8, Cal-1, Scene-3, WDBC-1, Mushroom-1 and Wine-2—in Figure 3.

As the feature-noise injection ratio rises from 0% to 40%, the AUC of every method declines, confirming that random noise interferes to varying degrees with both the feature representation and the learning of the detection boundary. Against the baselines, however, SMDL-SVDD retains a higher AUC at every noise level on all six tasks, and its performance degrades comparatively gently as noise increases. Its advantage remains pronounced even under the heavier noise perturbation of 30% and 40%, which indicates good robustness to random perturbation in the training data. Looking more closely, the traditional single-view methods SVDD, OCSVM and DW-SVDD degrade markedly as noise grows, which shows that learning a boundary directly on the raw features is readily disturbed by noisy dimensions. The semi-supervised methods SSAD, DeepSAD and AnoOnly improve detection by drawing on the additional supervisory information, but their performance also degrades to varying degrees as the level of noise perturbation increases. The multi-view methods MS-SVDD and mSVDD mitigate the effect to some extent by drawing on information from several views, but they still degrade noticeably at higher noise levels. SMDL-SVDD, by contrast, learns a more compact sparse representation through the synthesis and analysis dictionaries, which reduces the influence of redundant information and random perturbation on the anomaly boundary; the manifold regularisation constraint preserves the smoothness of the local geometric structure; and the cross-view consistency constraint exploits complementary information between views to weaken the effect of noise and bias in any single view. As a result the model still obtains a comparatively stable discriminative representation even when the training data are heavily perturbed by feature noise.

#### 4.4.3. Effect of the Labelled Normal Sample Ratio

To examine how the degree of annotation scarcity in the normal class affects performance, we vary the fraction of labelled normal samples in the training set. Because AnoOnly does not use the normal labels for explicit loss supervision, its training mechanism does not correspond directly to the labelled-normal ratio studied here, so only SSAD, DeepSAD and SMDL-SVDD are compared in this subsection. The ratio ρ is set in turn to 5%, 10%, 15%, 20%, 25%, 30%, 35% and 40%, with the data partitioning and parameter selection strategy being otherwise unchanged. We show four tasks drawn from datasets of different type and scale—WDBC-2, Wine-2, Handwritten-1 and Scene-4—in Figure 4.

The AUC of all three semi-supervised methods trends upward as the labelled normal ratio grows, which shows that more labelled normal samples provide fuller supervision for modelling the normal-class distribution. SMDL-SVDD improves the fastest as ρ rises from 5% to 25%, after which the increase in the AUC clearly slows: once about 25% of the normal samples are labelled, detection performance begins to saturate, and the marginal benefit of further normal-class annotation is limited. Notably, on all four tasks, the AUC of SMDL-SVDD at ρ=25% already exceeds the best results of SSAD and DeepSAD at ρ=40%, and even in the extreme case of ρ=5%, SMDL-SVDD remains the best method on all four tasks. Overall, SMDL-SVDD reaches good detection performance at a comparatively low normal-class annotation ratio and stabilises once that ratio reaches roughly 25%, which points to efficient use of labels and a tolerance for scarce normal-class annotation.

#### 4.4.4. Hyperparameter Analysis

To analyse how the key hyperparameters of SMDL-SVDD affect detection, we study the sensitivity to β, γ, δ and τ. Here β controls the strength of the $l_{2,1}$ regularisation on the sparse representation, γ balances the analysis-dictionary projection against the sparse codes, δ controls the representation consistency of unlabelled samples across views, and τ tunes the influence of the manifold regulariser that preserves local geometric structure. Following the settings of Section 4.3.1, each parameter is varied over $\left[10^{-3},10^{3}\right]$. When β and γ are analysed, δ and τ are held fixed, and vice versa. To cover different data scales, view sources and feature types, we select four tasks from both the naturally multi-view and the constructed multi-view data — Handwritten-4, Scene-2, WDBC-1 and Wine-3.

We first consider β and γ, with results in Figure 5. On WDBC-1 and Wine-3, the AUC stays at a high level across the combinations of β and γ and fluctuates only slightly, so SMDL-SVDD is fairly stable in these two parameters over a wide range. Handwritten-4 and Scene-2, by contrast, are markedly more sensitive to γ: the AUC is comparatively low when γ is small, and detection performance improves clearly and then stabilises as γ is increased appropriately. This indicates that strengthening the consistency between the analysis-dictionary projection and the sparse codes helps to obtain a more stable discriminative representation. For most values of γ, on the other hand, varying β has a relatively small effect on performance, which suggests a degree of parameter tolerance with respect to the strength of the sparse regulariser. A suitable setting of γ therefore improves the stability of representation learning, whereas β does not cause pronounced fluctuations over a wide range of values.

Turning to δ and τ, the results are shown in Figure 6. On Handwritten-4, WDBC-1 and Wine-3, the model maintains a high AUC over most parameter combinations, indicating good robustness to the cross-view consistency and manifold regularisation parameters. Scene-2 is notably more sensitive to δ: detection performance holds up, while δ stays in a moderate range, whereas for excessively large δ, the AUC drops appreciably at some values of τ. This suggests that an overly strong cross-view consistency constraint may force the views into representations that are too similar, eroding the complementary information they originally carried. It can also be observed that the value of τ modulates this behaviour to some extent, which indicates a degree of interaction between manifold regularisation and the cross-view consistency constraint. On tasks of this kind, the relationship between cross-view consistency and the preservation of manifold structure therefore has to be balanced with care.

Considering Figure 5 and Figure 6 together, SMDL-SVDD shows low sensitivity to the hyperparameters on tasks such as WDBC-1 and Wine-3 and also maintains good detection performance over a wide parameter range on Handwritten-4; Scene-2 shows greater sensitivity to some parameters and to their combinations, which indicates that different datasets place somewhat different demands on representation consistency, cross-view cooperation and the preservation of manifold structure. On the whole, SMDL-SVDD maintains comparatively stable detection performance under most parameter combinations and does not depend on a single, very narrow parameter window, although for some complex tasks, parameter selection still has to strike an appropriate balance between the different constraints.

#### 4.4.5. Convergence

To verify the convergence of the proposed algorithm, we track the objective value against the iteration count during training on four tasks—Handwritten-1, Cal-1, Wine-1 and Mushroom-1—with the iteration count on the horizontal axis and the normalised objective value on the vertical axis. As Figure 7 shows, the objective falls rapidly with iteration on all four datasets, levels off after roughly 20–30 iterations and changes little thereafter. This empirical behaviour agrees with the theoretical conclusion of Lemma 1 on monotone convergence of the objective sequence and shows that the optimisation algorithm converges stably in a small number of iterations.

#### 4.4.6. Ablation Study

To verify the contribution of manifold regularisation (MR) and the cross-view consistency constraint (CVC) to the detection performance of SMDL-SVDD, we carry out an ablation study in which the rest of the model structure and the experimental settings are left unchanged. Three ablated models are constructed: w/o MR & CVC removes manifold regularisation and the cross-view consistency constraint at the same time, that is, τ=0 and δ=0; w/o MR removes only the manifold regulariser, that is, τ=0, while keeping the cross-view consistency parameter δ of the full model; and w/o CVC removes only the cross-view consistency constraint, that is, δ=0, while keeping the manifold regularisation parameter τ of the full model. The complete SMDL-SVDD model retains both MR and CVC. Two tasks from the Caltech101-7 dataset are used in this subsection, and the mean AUC ± standard deviation is reported.

The results are given in Table 5. Both MR and CVC improve the detection performance of the model. Relative to the baseline in which both constraints are removed, introducing MR alone (w/o CVC) raises the AUC on Cal-1 and Cal-2 by 6.50 and 4.35 percentage points respectively, whereas introducing CVC alone (w/o MR) raises them by 2.13 and 0.43 percentage points; this indicates that manifold regularisation, by preserving the local geometric structure of the samples, has the more pronounced effect on the discriminative representation. Building on this, the complete model with both constraints raises the AUC further to the best value, which shows that the cross-view consistency constraint and manifold regularisation act complementarily.

#### 4.4.7. Training Time Comparison

Finally, to analyse computational cost, we compare the average training time of SMDL-SVDD with that of the baselines on Cal-1 and Mushroom-1. Figure 8 shows that SVDD and OCSVM train quickly, since both use a comparatively simple single-view one-class framework and learn the boundary directly in feature space without any joint representation optimisation. DeepSAD and AnoOnly, by contrast, need end-to-end feature learning and iterative optimisation through a deep neural network and therefore take considerably longer. The training time of SMDL-SVDD exceeds that of some traditional methods, mainly because it optimises the multi-view synthesis dictionary, analysis dictionary and sparse representations jointly while also accounting for manifold regularisation and cross-view consistency, which adds a certain amount of computation. Even so, SMDL-SVDD trains faster than DeepSAD and AnoOnly, and read together with the AUC results above, this indicates a reasonable balance between detection performance and computational efficiency.

## 5. Conclusions and Future Work

We have addressed anomaly detection under label scarcity and multi-view heterogeneity with SMDL-SVDD, a semi-supervised multi-view support vector data description framework built on dictionary learning. The method first learns sparse representations of the data in each view through a synthesis and an analysis dictionary, so as to obtain more discriminative features. It then admits a small number of labelled normal and anomalous samples into the SVDD hypersphere boundary modelling, and lets a large body of unlabelled samples take part in learning through manifold regularisation and a cross-view consistency constraint without assigning them any class label, thereby exploiting both the local geometric structure of the samples and the consistency and complementarity among views. The variables of the model are optimised iteratively by alternating convex search. Experimentally, SMDL-SVDD achieves the best AUC on 21 of 23 anomaly detection tasks, and the significance test together with the noise, label-ratio, hyperparameter, convergence, ablation and training-time studies indicate good detection performance, robustness, label efficiency and optimisation stability.

Two directions seem worth pursuing. One is an adaptive view-weighting mechanism that would adjust each view’s contribution dynamically according to its discriminative power and reliability. The other is efficient learning for online or streaming data, which would improve the adaptability of the model to complex dynamic data and its efficiency in practical deployment.

## Figures and Tables

**Figure 1:**
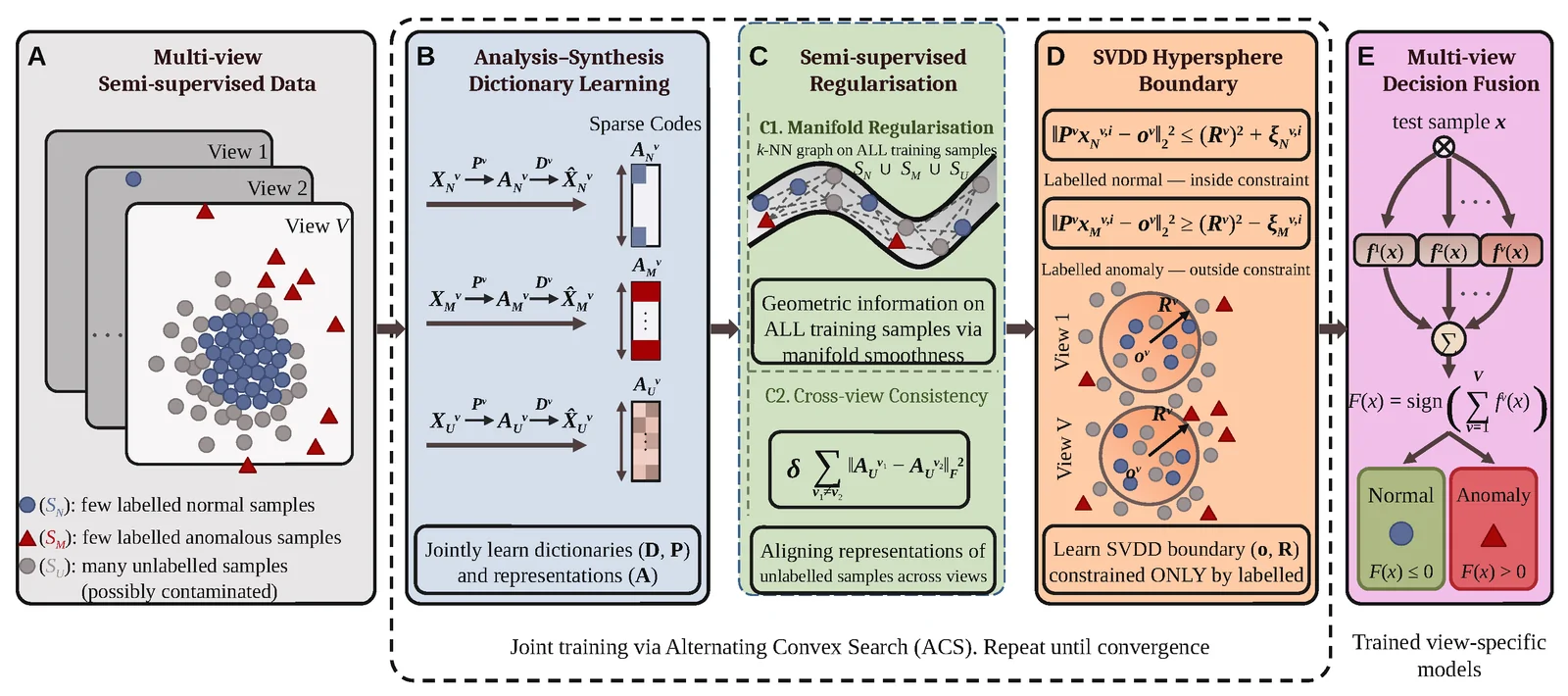
Overall framework of SMDL-SVDD, which integrates multi-view analysis–synthesis-dictionary learning, manifold regularisation, cross-view consistency for unlabelled samples, semi-supervised SVDD boundary learning and multi-view decision fusion for anomaly detection.

**Figure 2:**
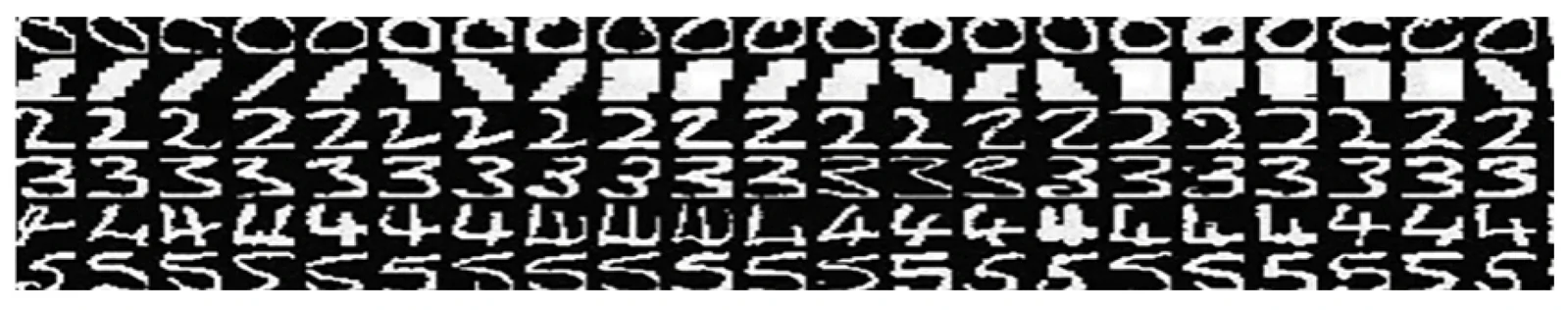
Sample images in the Handwritten dataset.

**Figure 3:**
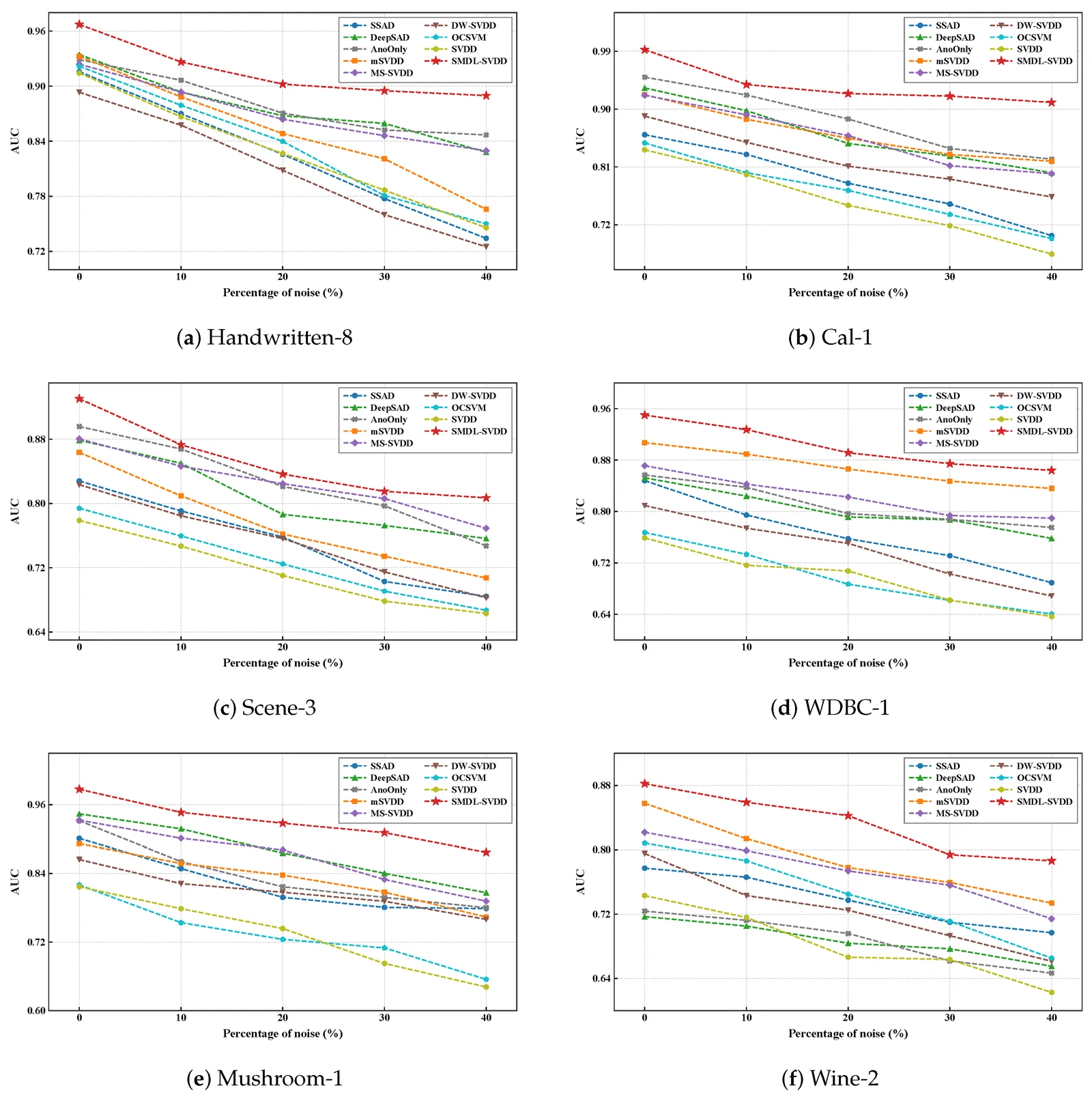
Effect of the ratio of feature-noise injection in the training data on the AUC of each method. Panels (**a**–**f**) correspond to Handwritten-8, Cal-1, Scene-3, WDBC-1, Mushroom-1 and Wine-2.

**Figure 4:**
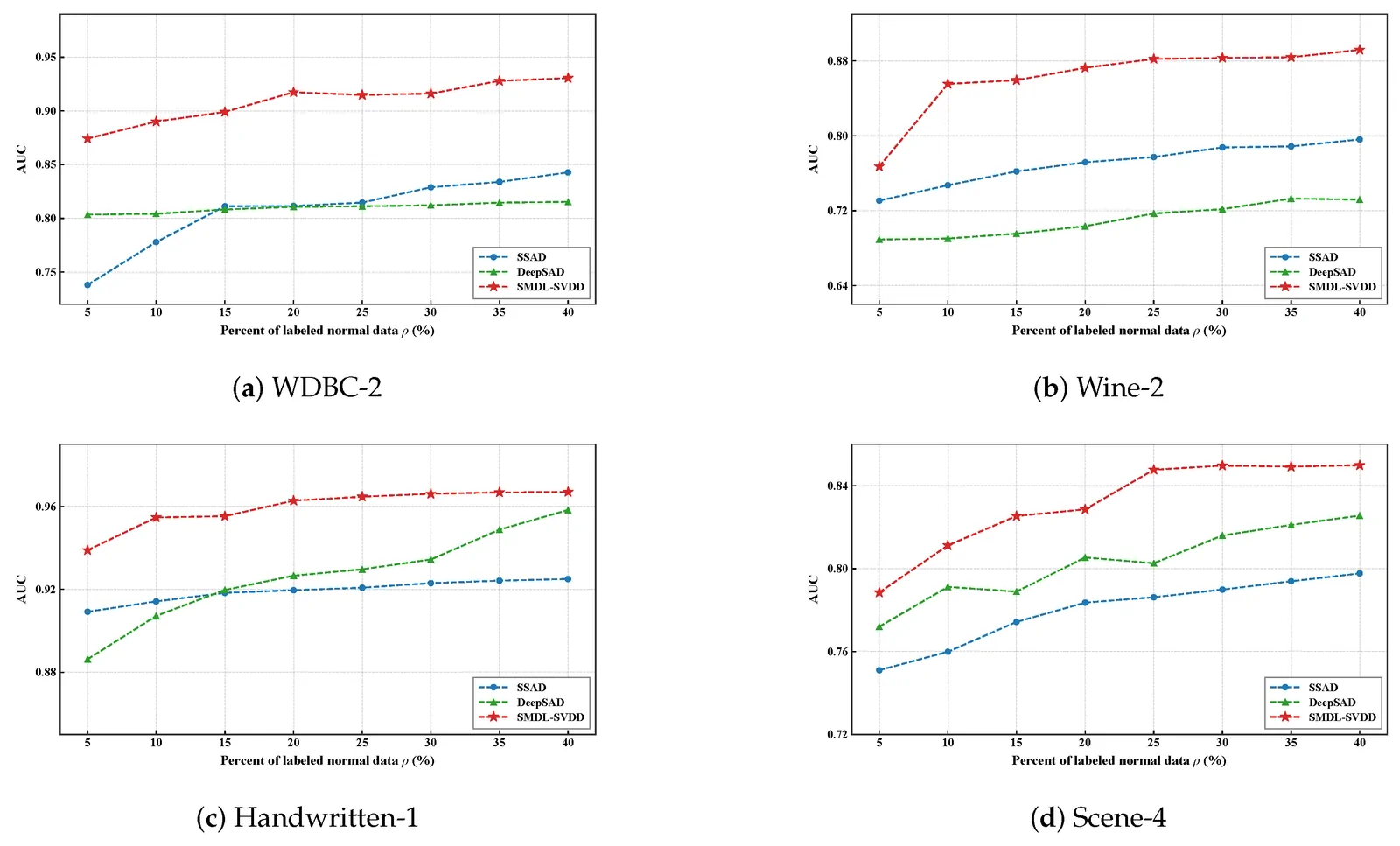
Effect of the labelled normal sample ratio ρ on the AUC of the three semi-supervised methods. Panels (**a**–**d**) correspond to WDBC-2, Wine-2, Handwritten-1 and Scene-4.

**Figure 5:**
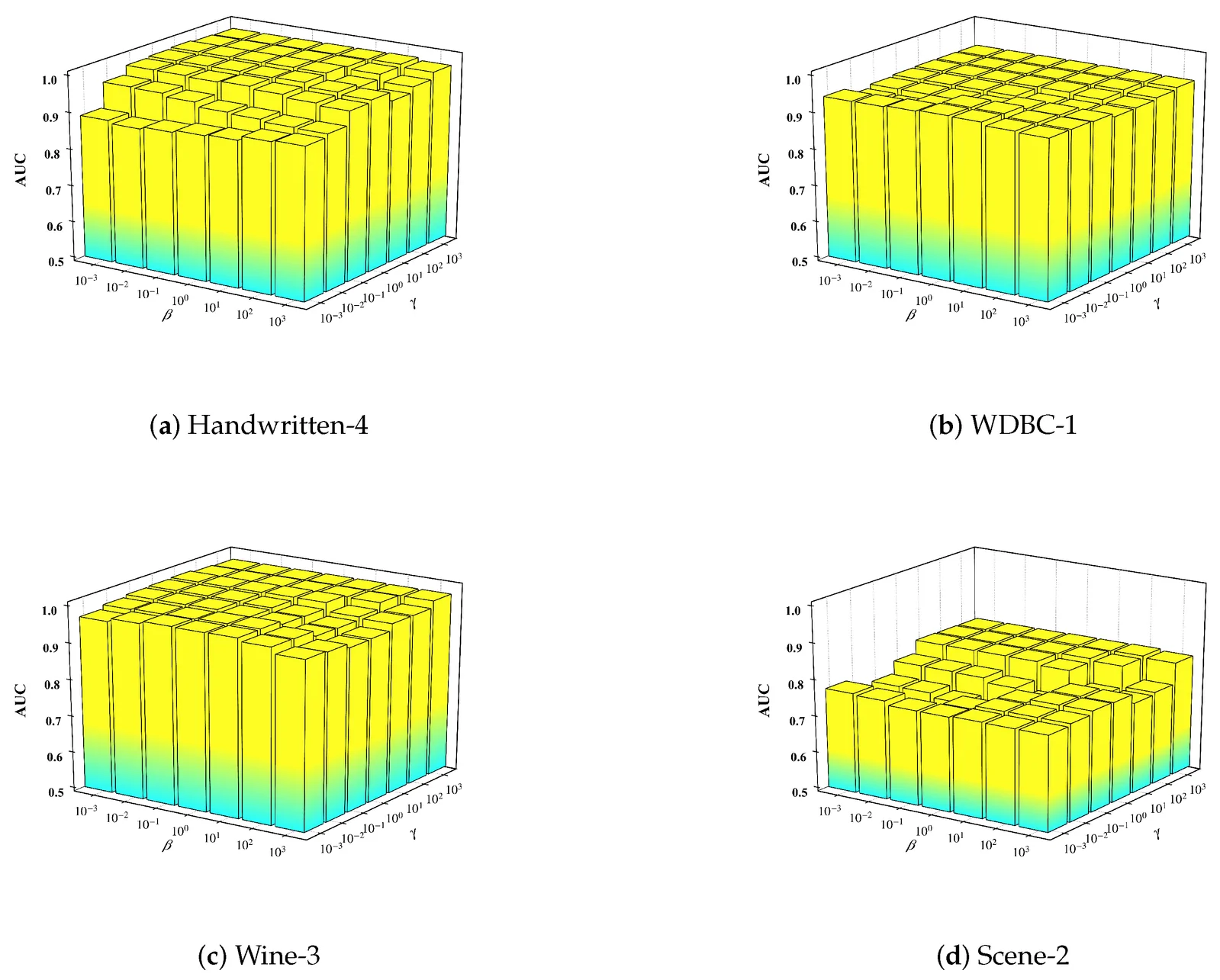
Effect of the hyperparameters β and γ on the detection performance (AUC) of SMDL-SVDD. Panels (**a**–**d**) correspond to Handwritten-4, WDBC-1, Wine-3 and Scene-2.

**Figure 6:**
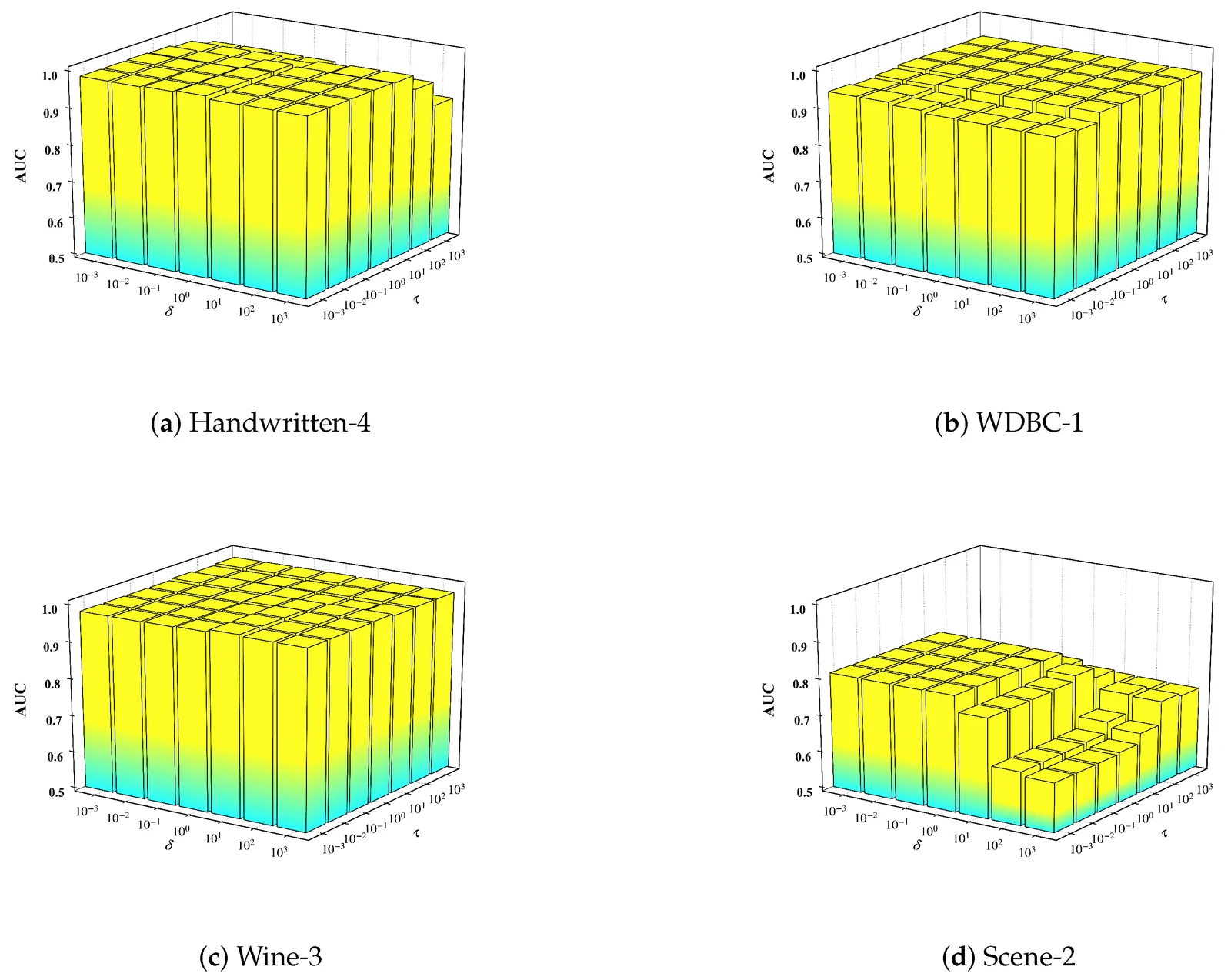
Effect of the hyperparameters δ and τ on the detection performance (AUC) of SMDL-SVDD. Panels (**a**–**d**) correspond to Handwritten-4, WDBC-1, Wine-3 and Scene-2.

**Figure 7:**
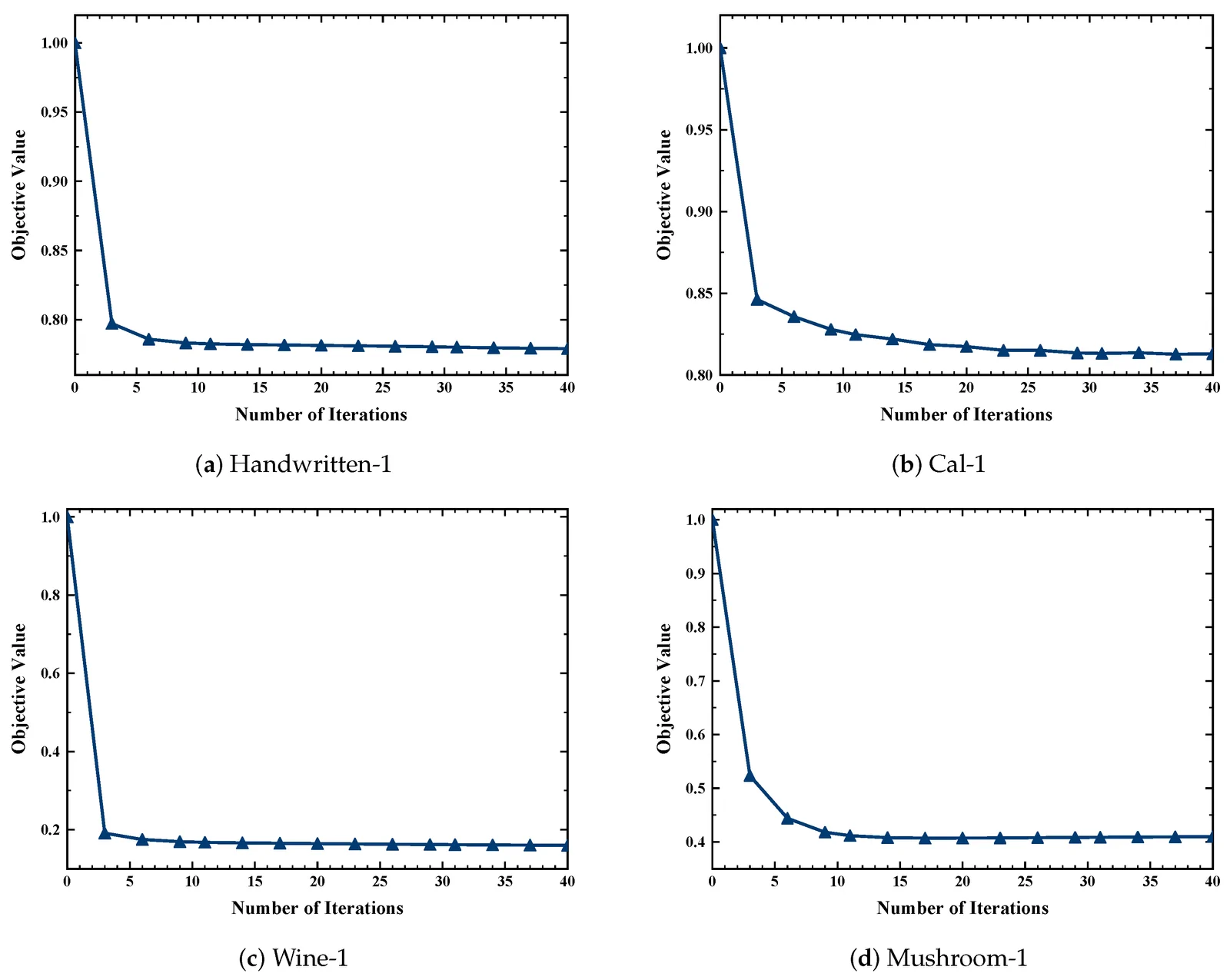
Convergence of SMDL-SVDD on different datasets. Panels (**a**–**d**) correspond to Handwritten-1, Cal-1, Wine-1 and Mushroom-1.

**Figure 8:**
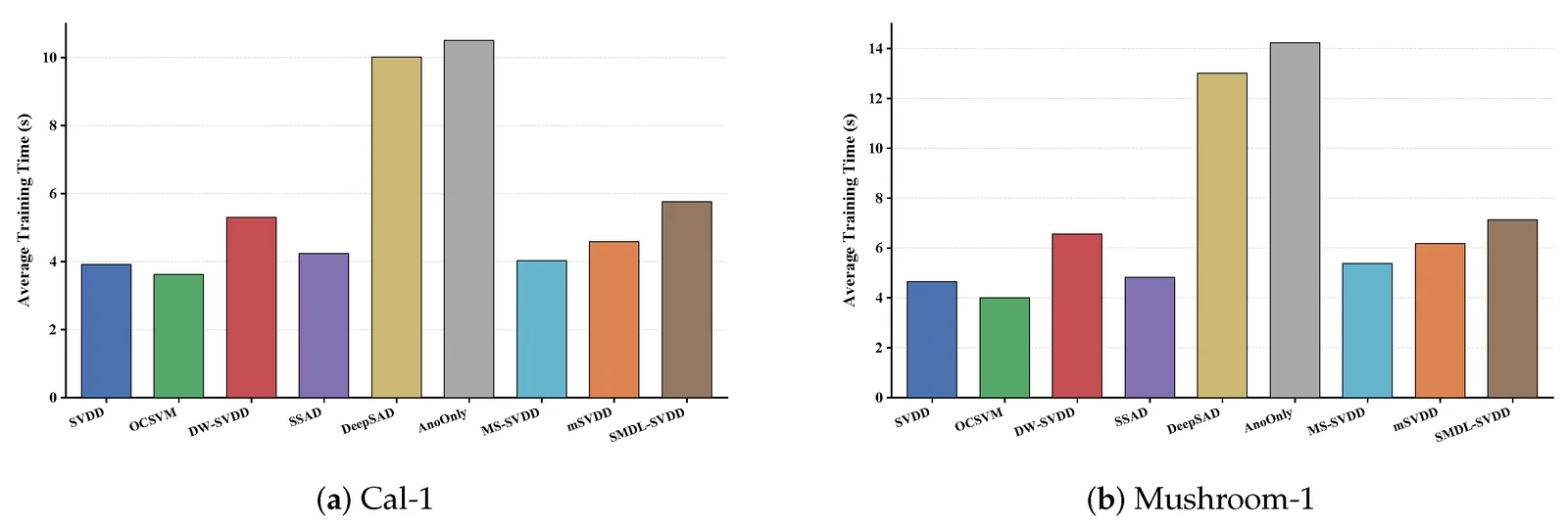
Average training time of the different methods on the Cal-1 and Mushroom-1 tasks.

**Table 1:** Statistics of the experimental datasets.

Dataset	Samples	Classes	Views	Dimension of Each View
Handwritten	2000	10	6	76, 216, 64, 240, 47, 6
Caltech101-7	1474	7	6	48, 40, 254, 1984, 512, 928
OutdoorScene	2688	8	4	512, 432, 256, 48
WDBC	569	2	3	10, 10, 10
Mushroom	8124	2	5	22, 27, 33, 22, 13
Wine	178	3	3	5, 4, 4

**Table 2:** Statistics of the 23 anomaly detection tasks.

Dataset	Class	Normal Samples	Anomalous Samples	Anomaly Ratio
Handwritten	Handwritten-0–Handwritten-9	200	20	9.09%
Caltech101-7	Cal-1	798	80	9.11%
	Cal-2	435	44	9.19%
OutdoorScene	Scene-1	410	41	9.09%
	Scene-2	374	37	9.00%
	Scene-3	360	36	9.09%
	Scene-4	356	36	9.18%
WDBC	WDBC-1	357	36	9.16%
	WDBC-2	212	21	9.01%
Mushroom	Mushroom-1	4208	421	9.09%
	Mushroom-2	3916	392	9.10%
Wine	Wine-1	59	24	28.92%
	Wine-2	71	28	28.28%
	Wine-3	48	19	28.36%

**Table 3:** Mean AUC ± standard deviation of the nine methods on the 23 anomaly detection tasks. The best result in each row is in bold.

Dataset	SVDD	OCSVM	DW-SVDD	SSAD	Deep SAD	Ano Only	MS-SVDD	mSVDD	SMDL-SVDD
WDBC-1	0.7589 ± 0.0441	0.7673 ± 0.0382	0.8092 ± 0.0322	0.8480 ± 0.0222	0.8524 ± 0.0661	0.8568 ± 0.0601	0.8713 ± 0.1011	0.9071 ± 0.0346	**0.9500 ± 0.0386**
WDBC-2	0.8018 ± 0.0774	0.8073 ± 0.0891	0.8039 ± 0.0593	0.8146 ± 0.0740	0.8112 ± 0.0840	0.8672 ± 0.0712	0.8604 ± 0.1116	0.8844 ± 0.0744	**0.9148 ± 0.0473**
Wine-1	0.8417 ± 0.0596	0.8611 ± 0.0805	0.8774 ± 0.0784	0.8944 ± 0.0829	0.8802 ± 0.0807	0.8254 ± 0.0999	0.9373 ± 0.0540	0.9317 ± 0.0762	**0.9786 ± 0.0222**
Wine-2	0.7432 ± 0.0712	0.8082 ± 0.0615	0.7955 ± 0.0487	0.7773 ± 0.1055	0.7170 ± 0.1413	0.7239 ± 0.1250	0.8216 ± 0.1093	0.8577 ± 0.0719	**0.8821 ± 0.0602**
Wine-3	0.7800 ± 0.0884	0.7433 ± 0.0667	0.7733 ± 0.0800	0.8607 ± 0.0516	0.8444 ± 0.1263	0.8189 ± 0.1216	0.9056 ± 0.0685	0.9286 ± 0.0586	**0.9917 ± 0.0151**
Mushroom-1	0.8169 ± 0.0043	0.8200 ± 0.0074	0.8644 ± 0.0472	0.9011 ± 0.0336	0.9439 ± 0.0083	0.9319 ± 0.0182	0.9328 ± 0.0308	0.8919 ± 0.0177	**0.9869 ± 0.0110**
Mushroom-2	0.8611 ± 0.0102	0.8515 ± 0.0080	0.8661 ± 0.0463	0.9137 ± 0.0191	0.9672 ± 0.0102	0.9334 ± 0.0199	0.9071 ± 0.0495	0.9304 ± 0.0244	**0.9738 ± 0.0080**
Handwritten-0	0.9217 ± 0.0570	0.9133 ± 0.0417	0.9183 ± 0.0213	0.9292 ± 0.0245	**0.9797 ± 0.0233**	0.9714 ± 0.0328	0.9550 ± 0.0586	0.9563 ± 0.0467	0.9652 ± 0.0335
Handwritten-1	0.9175 ± 0.0313	0.9239 ± 0.0327	0.9275 ± 0.0171	0.9208 ± 0.0176	0.9297 ± 0.0407	0.9492 ± 0.0525	0.9467 ± 0.0297	0.9482 ± 0.0424	**0.9647 ± 0.0424**
Handwritten-2	0.8983 ± 0.0276	0.9025 ± 0.0294	0.9250 ± 0.0296	0.9092 ± 0.0234	0.9392 ± 0.0338	0.9103 ± 0.0491	0.9233 ± 0.0396	0.9303 ± 0.0445	**0.9878 ± 0.0121**
Handwritten-3	0.8912 ± 0.0496	0.8881 ± 0.0305	0.9050 ± 0.0208	0.9272 ± 0.0251	0.9031 ± 0.0519	0.9481 ± 0.0517	0.9058 ± 0.0609	0.9214 ± 0.0593	**0.9678 ± 0.0295**
Handwritten-4	0.9025 ± 0.0361	0.9117 ± 0.0264	0.9167 ± 0.0204	0.9225 ± 0.0194	0.9519 ± 0.0380	0.9417 ± 0.0436	0.9450 ± 0.0385	0.9417 ± 0.0463	**0.9897 ± 0.0106**
Handwritten-5	0.8783 ± 0.0379	0.8767 ± 0.0318	0.8967 ± 0.0287	0.8758 ± 0.0126	0.9017 ± 0.0573	0.9508 ± 0.0394	0.9139 ± 0.0507	0.9044 ± 0.0672	**0.9603 ± 0.0405**
Handwritten-6	0.9067 ± 0.0353	0.9108 ± 0.0356	0.9333 ± 0.0171	0.9067 ± 0.0162	0.9506 ± 0.0411	0.9425 ± 0.0440	0.9406 ± 0.0470	0.9544 ± 0.0655	**0.9836 ± 0.0247**
Handwritten-7	0.8950 ± 0.0467	0.9100 ± 0.0385	0.9083 ± 0.0211	0.9483 ± 0.0196	0.9744 ± 0.0218	0.9842 ± 0.0173	0.9469 ± 0.0641	0.9454 ± 0.0513	**0.9864 ± 0.0251**
Handwritten-8	0.9142 ± 0.0298	0.9217 ± 0.0282	0.8933 ± 0.0355	0.9158 ± 0.0237	0.9344 ± 0.0396	0.9289 ± 0.0404	0.9239 ± 0.0427	0.9325 ± 0.0717	**0.9672 ± 0.0461**
Handwritten-9	0.9033 ± 0.0371	0.8983 ± 0.0497	0.9042 ± 0.0321	0.9175 ± 0.0336	0.9686 ± 0.0219	0.9581 ± 0.0391	0.9236 ± 0.0737	0.9461 ± 0.0455	**0.9897 ± 0.0105**
Cal-1	0.8367 ± 0.0313	0.8473 ± 0.0204	0.8890 ± 0.0094	0.8601 ± 0.0228	0.9329 ± 0.0267	0.9497 ± 0.0210	0.9216 ± 0.0346	0.9224 ± 0.0213	**0.9924 ± 0.0042**
Cal-2	0.8370 ± 0.0408	0.8244 ± 0.0355	0.8401 ± 0.0204	0.8658 ± 0.0837	0.9022 ± 0.0411	0.9575 ± 0.0456	0.9230 ± 0.0307	0.9441 ± 0.0430	**0.9915 ± 0.0158**
Scene-1	0.7447 ± 0.0815	0.7618 ± 0.0445	0.7644 ± 0.0522	0.7735 ± 0.0302	0.7878 ± 0.0973	0.8065 ± 0.0743	0.7728 ± 0.0868	**0.8416 ± 0.0490**	0.8236 ± 0.0871
Scene-2	0.7937 ± 0.0995	0.7758 ± 0.0341	0.7708 ± 0.0532	0.7890 ± 0.0949	0.7928 ± 0.0809	0.8130 ± 0.0771	0.7718 ± 0.1000	0.7951 ± 0.0776	**0.8168 ± 0.0716**
Scene-3	0.7789 ± 0.0274	0.7940 ± 0.0462	0.8234 ± 0.0322	0.8280 ± 0.0236	0.8787 ± 0.0404	0.8958 ± 0.0674	0.8804 ± 0.0468	0.8636 ± 0.0317	**0.9304 ± 0.0466**
Scene-4	0.7758 ± 0.0555	0.7720 ± 0.0484	0.7975 ± 0.0411	0.7862 ± 0.0676	0.8026 ± 0.0535	0.8054 ± 0.0722	0.8074 ± 0.0556	0.8184 ± 0.0473	**0.8477 ± 0.0530**

**Table 4:** Wilcoxon signed-rank test of SMDL-SVDD against each baseline.

Comparisons	SVDD	OCSVM	DW-SVDD	SSAD	DeepSAD	AnoOnly	MS-SVDD	mSVDD
$R^{+}$	276	276	276	276	273	273	276	273
$R^{-}$	0	0	0	0	3	3	0	3
*p*-value	2.384 × 10^−7^	2.384 × 10^−7^	2.384 × 10^−7^	2.384 × 10^−7^	1.192 × 10^−6^	1.192 × 10^−6^	2.384 × 10^−7^	1.192 × 10^−6^

**Table 5:** Ablation results for the different components of SMDL-SVDD. The best result in each column is shown in bold.

Variant	MR	CVC	Cal-1	Cal-2
w/o MR & CVC	×	×	0.9155 ± 0.0165	0.9381 ± 0.0343
w/o MR	×	✓	0.9368 ± 0.0167	0.9424 ± 0.0310
w/o CVC	✓	×	0.9805 ± 0.0068	0.9816 ± 0.0182
**SMDL-SVDD**	✓	✓	**0.9924 ± 0.0042**	**0.9915 ± 0.0158**

## Data Availability

All six datasets used in this study are publicly available. Handwritten (Multiple Features), WDBC (Wisconsin Diagnostic Breast Cancer), Mushroom and Wine are available from the UCI Machine Learning Repository at https://archive.ics.uci.edu/ (accessed on 21 September 2026); Caltech101-7 is the standard seven-class subset of the Caltech101 dataset; and OutdoorScene is the public outdoor scene image dataset. No new data were created in this study.
